# Expression of the Calcitonin Receptor-like Receptor (CALCRL) in Normal and Neoplastic Tissues

**DOI:** 10.3390/ijms24043960

**Published:** 2023-02-16

**Authors:** Benjamin Wende, Anna-Sophia Liselott Beyer, Niklas Ruhnke, Daniel Kaemmerer, Jörg Sänger, Stefan Schulz, Amelie Lupp

**Affiliations:** 1 Institute of Pharmacology and Toxicology, Jena University Hospital, 07747 Jena, Germany; 2 Department of General and Visceral Surgery, Zentralklinik Bad Berka, 99438 Bad Berka, Germany; 3 Laboratory of Pathology and Cytology Bad Berka, 99438 Bad Berka, Germany

**Keywords:** calcitonin receptor-like receptor, calcitonin gene-related peptide, calcitonin gene-related peptide receptor, adrenomedullin, adrenomedullin receptor, amylin, amylin receptor, receptor activity-modifying protein, antibody, immunohistochemistry, tumours

## Abstract

Little information is available concerning protein expression of the calcitonin receptor-like receptor (CALCRL) at the protein level. Here, we developed a rabbit monoclonal antibody, 8H9L8, which is directed against human CALCRL but cross-reacts with the rat and mouse forms of the receptor. We confirmed antibody specificity via Western blot analyses and immunocytochemistry using the CALCRL-expressing neuroendocrine tumour cell line BON-1 and a CALCRL-specific small interfering RNA (siRNA). We then used the antibody for immunohistochemical analyses of various formalin-fixed, paraffin-embedded specimens of normal and neoplastic tissues. In nearly all tissue specimens examined, CALCRL expression was detected in the capillary endothelium, smooth muscles of the arterioles and arteries, and immune cells. Analyses of normal human, rat, and mouse tissues revealed that CALCRL was primarily present in distinct cell populations in the cerebral cortex; pituitary; dorsal root ganglia; epithelia, muscles, and glands of the larger bronchi; intestinal mucosa (particularly in enteroendocrine cells); intestinal ganglia; exocrine and endocrine pancreas; arteries, capillaries, and glomerular capillary loops in the kidneys; the adrenals; Leydig cells in the testicles; and syncytiotrophoblasts in the placenta. In the neoplastic tissues, CALCRL was predominantly expressed in thyroid carcinomas, parathyroid adenomas, small-cell lung cancers, large-cell neuroendocrine carcinomas of the lung, pancreatic neuroendocrine neoplasms, renal clear-cell carcinomas, pheochromocytomas, lymphomas, and melanomas. In these tumours with strong expression of CALCRL, the receptor may represent a useful target structure for future therapies.

## 1. Introduction

The peptide hormones calcitonin, calcitonin gene-related peptide (CGRP), adrenomedullin, adrenomedullin 2, and amylin are, structurally, closely related peptides. They mediate their effects through a family of G protein-coupled receptors comprising the calcitonin receptor (CTR), the calcitonin gene-related peptide receptor (CGRPR), the adrenomedullin receptors 1 and 2 (AM_1_ receptor; AM_2_ receptor), and the amylin receptors 1, 2, and 3 (AMY_1_ receptor, AMY_2_ receptor, and AMY_3_ receptor), all of which are also able to bind the other ligands [1,2,3]. With the only exception of the CTR, these receptors consist of two components: The CTR or the calcitonin receptor-like receptor (CALCRL, also known as CRLR) and one of three receptor activity-modifying proteins (RAMP1, RAMP2, or RAMP3). The various combinations constitute the CGRPR (CALCRL + RAMP1), the AM_1_ receptor or AM_2_ receptor (CALCRL + RAMP2 or RAMP3), and the AMY_1_ receptor, AMY_2_ receptor, or AMY_3_ receptor (CTR + RAMP1, RAMP2, or RAMP3) [1,2,3]. Peptides in the calcitonin family and their receptors are involved in numerous activities, including the regulation of blood pressure and heart function, the release of hormones from the pituitary gland, the maintenance of various homeostatic processes (e.g., body temperature, food intake, blood sugar, and calcium levels), and the functions of the gastrointestinal tract. These peptides and their receptors play important roles in the pathophysiology of migraine and pain, various cardiovascular and renal disorders, diabetes mellitus, osteoporosis, and numerous inflammatory diseases including sepsis [1,4,5,6,7,8,9,10]. Notably, salmon calcitonin has been used for decades as a therapeutic agent for postmenopausal osteoporosis, Paget’s disease, and other painful bone diseases [11]. Additionally, antibodies against CGRP or CGRPR (e.g., eptinezumab, fremanezumab, galcanezumab, and erenumab), as well as small-molecule CGRPR antagonists (“gepants”, e.g., atogepant, rimegepant, and ubrogepant), are available for the prevention or the therapy of migraines, respectively [8,10,12]. Pramlintide, an AMY analogue, was approved by the United States Food and Drug Administration in 2005 for patients with type 1 or type 2 diabetes mellitus who use insulin [13]. Moreover, there is increasing evidence that adrenomedullin and its receptors are associated with the development and spread of tumours, resulting in the proposal of blockades for adrenomedullin and its receptors as potential treatments in multiple cancers [7,9,14].

As noted above, the clinical importance of the calcitonin family peptides has attracted increasing interest in recent years. However, apart from a detailed study of the expression of the GCRPR in trigeminal ganglia and cerebral vessels [15,16,17,18,19,20,21,22,23,24] and some studies on the presence of the GCRPR also in other regions of the central and peripheral nervous system, including diverse brain areas, spinal cord, and retina [18,25,26,27,28,29,30,31] and in other vascular areas [32], only few data are available on CALCRL, CGRPR, and adrenomedullin receptors in terms of protein expression both in healthy and diseased peripheral tissues, presumably because no suitable antibodies have been available.

Given the numerous advantages of monoclonal antibodies over polyclonal ones, we developed a rabbit monoclonal antibody, 8H9L8, directed against an N-terminal sequence in human CALCRL. The antibody was first tested for its specificity using CALCRL-expressing BON-1 cells, as well as a CALCRL-specific small interfering RNA (siRNA). We then used the antibody for immunohistochemical analyses of diverse formalin-fixed, paraffin-embedded normal and neoplastic human tissue specimens to establish a broad expression profile for CALCRL both for normal and neoplastic human tissues. Because the antibody cross-reacts with the rat and mouse forms of CALCRL, we also conducted analyses of the CALCRL staining patterns in various normal rat and mouse tissues in order to detect possible species differences. Finally, to obtain additional information regarding the CALCRL/RAMP isoform combinations (i.e., members of the CTR family) present in our tissue specimens, we conducted double-labelling immunohistochemistry analyses of CALCRL and each of the RAMP proteins in a set of CALCRL-positive normal and neoplastic human tissue samples.

Overall, we pursued three main goals with the present work, namely, (1) a thorough characterization of the novel antibody, (2) the creation of a CALCRL expression profile for normal (human) tissues, and (3) the creation of a CALCRL expression profile for human tumours.

## 2. Results

### 2.1. Evaluation of the Specificity of the Rabbit Anti-Human CALCRL Antibody

The specificity of the anti-CALCRL antibody 8H9L8 was first tested by Western blot analyses and immunocytochemistry in BON-1 cells, which endogenously express CALCRL.

**Figure 1:**
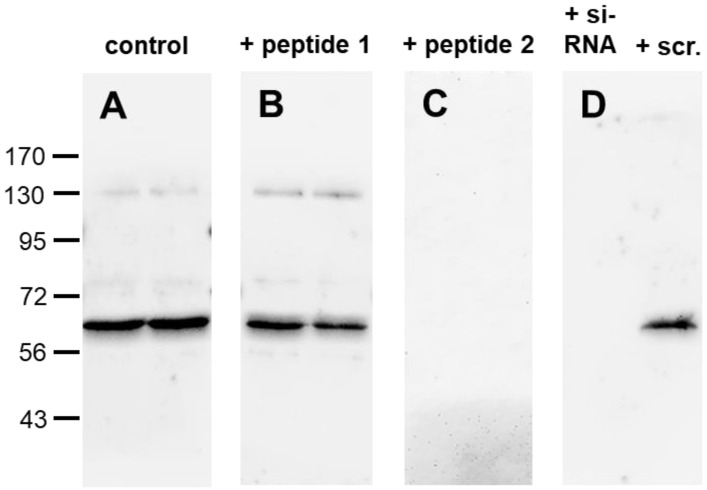
Specificity analysis of the rabbit monoclonal anti-human calcitonin receptor-like receptor (CALCRL) antibody 8H9L8 by Western blot analyses. (**A**) Western blot analysis of membrane preparations of BON-1 cells that endogenously express CALCRL. To establish controls for adsorption, the antibody 8H9L8 was preincubated for 2 h with either 10 µg/mL of a control peptide that corresponded to a different region of the receptor (peptide 1) (**B**) or 10 µg/mL of the immunising peptide (peptide 2) (**C**). (**D**) Western blot analysis of membrane preparations of BON-1 cells after transfection with siRNA targeting CALCRL (left lane) or with a scrambled siRNA (scr., right lane). Ladder indicates migration of protein molecular weight markers (kDa) and is the same for all displayed blots. All results are representative of three independent experiments.

When membrane preparations from BON-1 cells were electrophoretically separated and immunoblotted, the antibody recognised a strong band that migrated at approximately 66 kDa (Figure 1A). A weak band was additionally observed at approximately 130 kDa, most likely representing a receptor dimer, and a very faint band at approximately 82 kDa likely corresponding to RAMP-coupled CALCRL (with RAMP molecular weights ranging from approximately 16.5 to 20 kDa). After preincubation of the antibody with the control peptide (peptide 1), the signal intensities remained unchanged (Figure 1B). By contrast, after preadsorption with the immunising peptide (peptide 2), the immunoreactive bands were completely absent (Figure 1C). Similarly, when CALCRL expression was silenced with a CALCRL-specific siRNA, the immunoreactive band was completely extinguished (Figure 1D, left lane), whereas after transfection of the cells with a scrambled siRNA, the immunosignal remained unchanged (Figure 1D, right lane).

**Figure 2:**
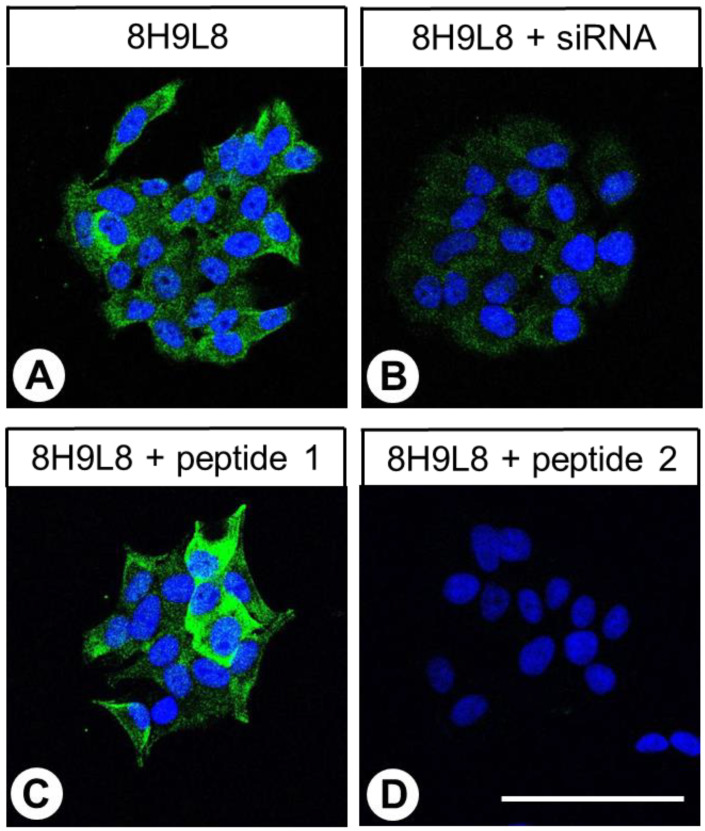
Specificity analysis of the rabbit monoclonal anti-human calcitonin receptor-like receptor (CALCRL) antibody 8H9L8 by immunocytochemistry. (**A**) BON-1 cells that endogenously express CALCRL were fixed and stained with the anti-CALCRL antibody 8H9L8, followed by an Alexa Fluor 488-conjugated anti-rabbit secondary antibody. (**B**) To analyse antibody specificity, CALCRL expression was silenced in BON-1 cells using a CALCRL-specific siRNA. (**C**) To establish controls for adsorption, the anti-CALCRL antibody was preincubated for 2 h with either 10 µg/mL of a control peptide (peptide 1) or (**D**) 10 µg/mL of the immunising peptide (peptide 2). Green colour represents CALCRL; blue colour represents 4′,6-diamidino-2-phenylindole (DAPI)-stained DNA. Scale bar, 100 µm (**A**–**D**). All results are representative of three independent experiments.

Immunocytochemistry analyses revealed that the receptor was present both in the cytoplasm and at the cell membrane of untreated BON-1 cells (Figure 2A). When CALCRL expression was silenced using a CALCRL-specific siRNA, the immunostaining intensity was substantially reduced (Figure 2B). Additionally, in contrast to the results after preincubation with the control peptide (peptide 1, Figure 2C), preincubation of the antibody 8H9L8 with the immunising peptide (peptide 2) led to the complete absence of receptor immunostaining (Figure 2D).

**Figure 3:**
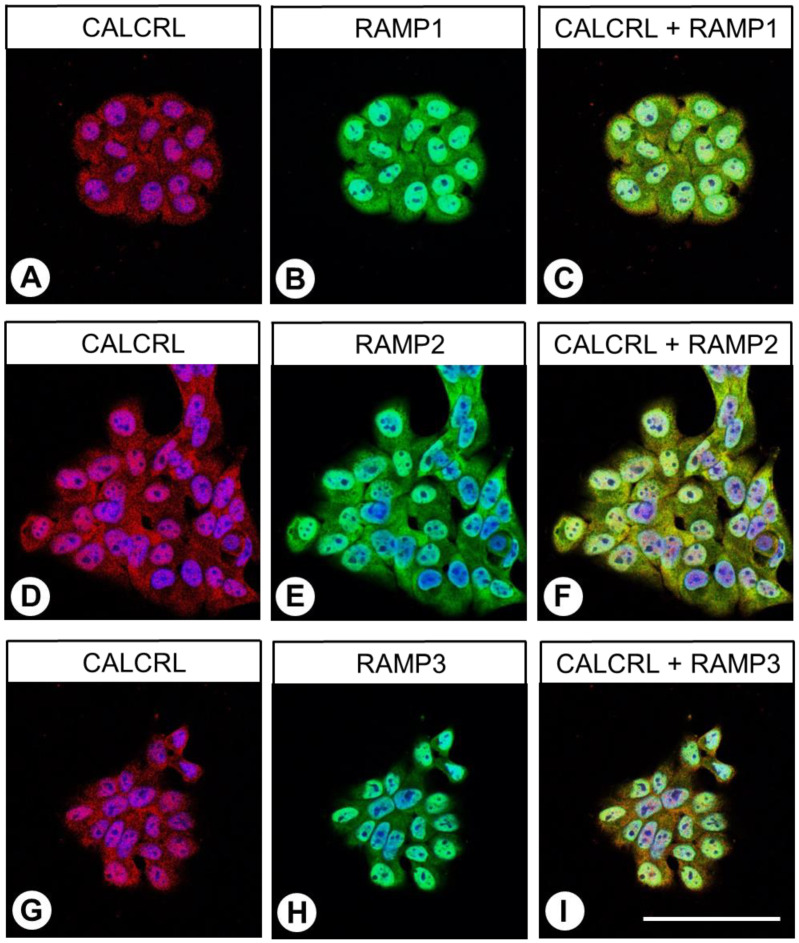
Double-labelling immunocytochemical analysis of calcitonin receptor-like receptor (CALCRL) expression (**A**,**D**,**G**) and the expression of receptor activity-modifying protein (RAMP) 1 (**B**), RAMP2 (**E**), or RAMP3 (**H**) in BON-1 cells. Labelling of CALCRL was visualised using Cy3-conjugated anti-rabbit antibody (red). Labelling of RAMP1, RAMP2, or RAMP3 was visualised using Alexa Fluor 488-conjugated rabbit anti-RAMP1, -RAMP2, or -RAMP3 antibody (green). Overlapping expression is represented by orange/yellow colour (**C**,**F**,**I**). Blue colour represents 4′,6-diamidino-2-phenylindole (DAPI)-stained DNA. Scale bar: 100 µm.

Subsequent immunocytochemistry double-labelling experiments revealed the presence of both RAMP1 and RAMP2, as well as low expression of RAMP3, in BON-1 cells, and all three proteins were clearly co-expressed with CALCRL (Figure 3).

### 2.2. Immunohistochemical Detection of CALCRL Expression in Normal Human, Rat, and Mouse Tissues

**Figure 4:**
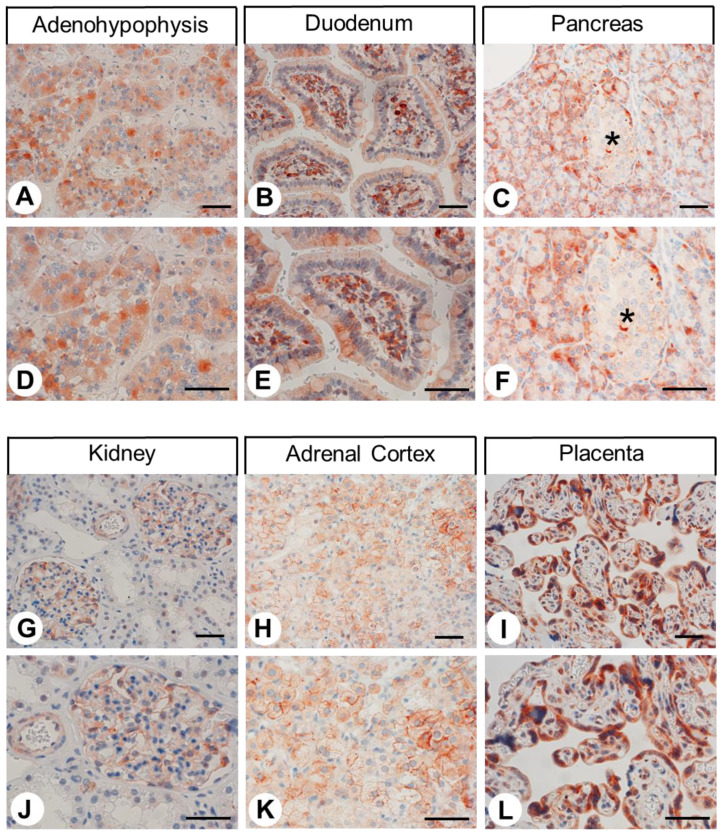
Immunohistochemical detection of CALCRL localisation in normal human tissues. Immunohistochemical staining (red-brown colour) and counterstaining with haematoxylin. (**D**,**E**,**F**) and (**J**,**K**,**L**) represent enlarged sections of (**A**,**B**,**C**) and (**G**,**H**,**I**). Asterisk in (**C**,**F**): Pancreatic islet. Scale bars, 50 µm.

**Figure 5:**
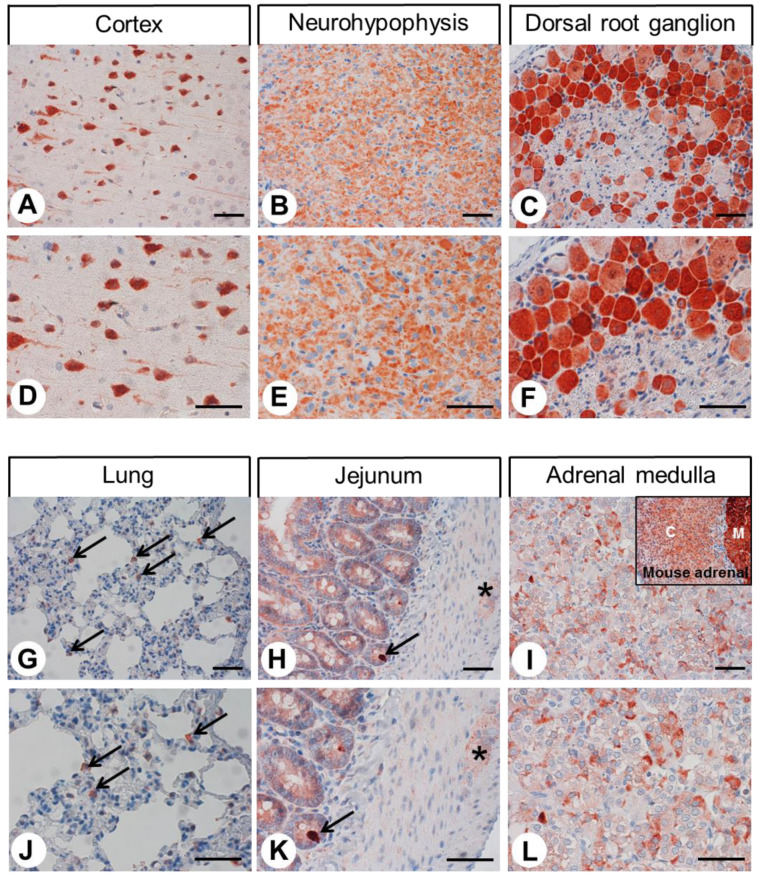
Immunohistochemical detection of CALCRL localisation in normal rat tissues. Immunohistochemical staining (red-brown colour) and counterstaining with haematoxylin. (**D**,**E**,**F**) and (**J**,**K**,**L**) represent enlarged sections of (**A**,**B**,**C**) and (**G**,**H**,**I**). Arrows in (**G**,**J**): Positive alveolar macrophages; arrow in (**H**,**K**): Enteroendocrine cell; asterisk in (**H**,**K**): Enteric ganglion; inset in (**I**): Mouse adrenal gland. C in (**I**): Adrenal cortex; M in (**I**): Adrenal medulla. Scale bars, 50 µm.

Next, we used the rabbit monoclonal anti-CALCRL antibody 8H9L8 for immunohistochemical staining of various normal human tissues. Some examples of positive staining patterns are shown in Figure 4 (adenohypophysis, duodenum, pancreas, kidney, adrenal cortex, and placenta, respectively). The immunostaining patterns in normal rat and mouse tissues were very similar to the findings in human tissues. Complementary examples of the positive staining patterns in rat tissues to the staining in human tissues shown in Figure 4 are depicted in Figure 5 (cortex, neurohypophysis, dorsal root ganglion, lung, jejunum, and adrenal medulla, respectively). Only the adrenal medulla exhibited stronger staining in mouse tissues than in the corresponding rat and human tissues (see inset in Figure 5I), indicating some species differences here. Both cytoplasmic and membranous staining patterns were observed in all cases. Regardless of any staining of organ- or tissue-specific cells, nearly all normal and neoplastic specimens exhibited strong immunostaining in capillary endothelia, the muscular layers of arterioles and arteries, and immune cells (with morphologies suggestive of monocytes or macrophages). In normal human, rat, and mouse tissues, strong CALCRL expression was present in distinct cell populations in the cerebral cortex (Figure 5A,D), which presumably represented both pyramidal cells and glial cells. Varying degrees of CALCRL expression were present in all cell types in the anterior pituitary gland (Figure 4A,D); the nerve fibres and Herring bodies in the posterior pituitary (Figure 5B,E); all ganglion cells in the dorsal root ganglia (most intensely in small and medium-sized cells) (Figure 5C,F); the intestinal mucosa (Figure 4B,E and Figure 5H,K), with intense staining of neuroendocrine cells (Figure 5H,K, arrow) and intestinal ganglia (Figure 5H,K, asterisk); the exocrine and endocrine pancreas (most intensely in a peripherally located subpopulation of islet cells) (Figure 4C,F); the arteries, capillaries, and glomerular capillary loops in the kidneys that were otherwise CALCRL-negative (Figure 4G,J); the adrenal medulla (Figure 5I,L) and all three layers of the adrenal cortex (Figure 4H,K); the Leydig cells in the testicles that were otherwise receptor-negative (not shown); and the placental syncytiotrophoblasts (Figure 4I,L). By contrast, no immunostaining was observed (except for a few cells that presumably represented macrophages) in the thymus, spleen, lymph nodes, heart, or liver (not shown). Strong CALCRL staining was also present in alveolar macrophages (Figure 5G,J, arrows) and the epithelium, muscles, and bronchial glands of larger bronchi. The remaining portions of lung tissue were CALCRL-negative (Figure 5G,J).

**Figure 6:**
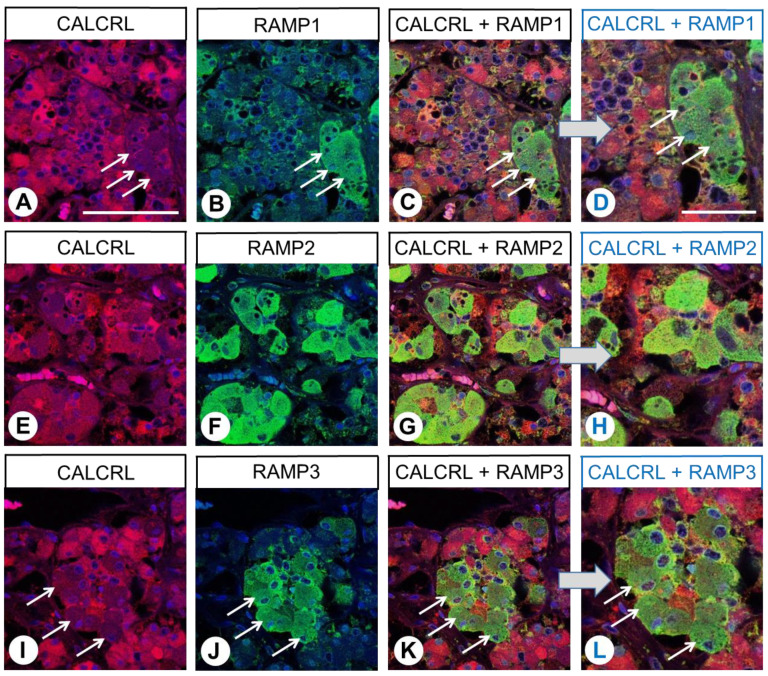
Double-labelling immunohistochemical analysis of calcitonin receptor-like receptor (CALCRL) expression (**A**,**E**,**I**) and the expression of receptor activity-modifying protein (RAMP) 1 (**B**), RAMP2 (**F**), or RAMP3 (**J**) in human pituitary tissue. Labelling of CALCRL was visualised using Cy3-conjugated anti-rabbit antibody (red). Labelling of RAMP1, RAMP2, or RAMP3 was visualised using Alexa Fluor 488-conjugated rabbit anti-RAMP1, -RAMP2, or -RAMP3 antibody (green). Overlapping expression is represented by orange/yellow colour (**C**,**D**,**G**,**H**,**K**,**L**). Blue colour represents 4′,6-diamidino-2-phenylindole (DAPI)-stained DNA. Arrows: CALCRL-negative cells that express RAMP1 or RAMP3. (**D**,**H**,**L**) represent enlarged sections of (**C**,**G**,**K**). Scale bar: 100 µm (**A**–**C**,**E**–**G**,**I**–**K**); 50 µm (**D**,**H**,**L**).

Subsequently, immunofluorescence double-labelling experiments were performed on human pituitary, duodenum, pancreas, adrenal cortex, and placenta specimens. Most cells in the anterior pituitary showed clear CALCRL expression, although the intensity differed (Figure 6A,E,I). By contrast, RAMP1, RAMP2, and RAMP3 were each only found in certain cell populations, and the immunofluorescence in cells expressing RAMP2 was more prominent than that in cells expressing either RAMP1 or RAMP3 (Figure 6B,F,J). Furthermore, co-expression with CALCRL was primarily found for RAMP2 (Figure 6G,H), whereas RAMP1- and RAMP3-positive cells mostly showed no CALCRL expression (white arrows in Figure 6A–D,I–L).

**Figure 7:**
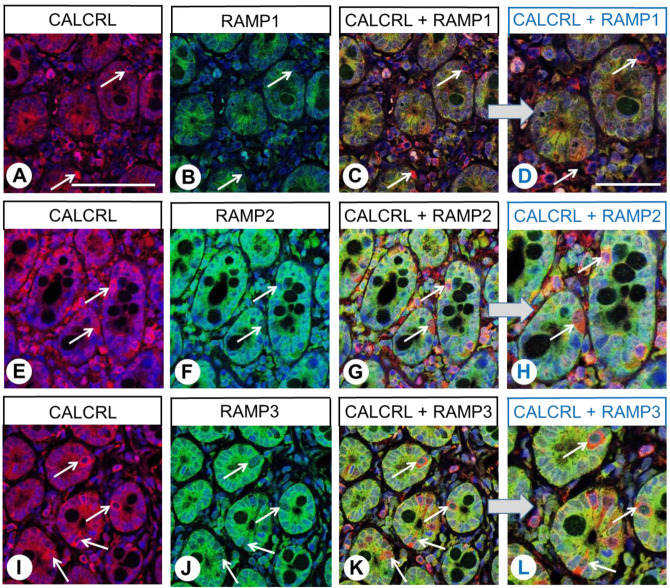
Double-labelling immunohistochemical analysis of calcitonin receptor-like receptor (CALCRL) expression (**A**,**E**,**I**) and the expression of receptor activity-modifying protein (RAMP) 1 (**B**), RAMP2 (**F**), or RAMP3 (**J**) in human duodenal tissue. Labelling of CALCRL was visualised using Cy3-conjugated anti-rabbit antibody (red). Labelling of RAMP1, RAMP2, or RAMP3 was visualised using Alexa Fluor 488-conjugated rabbit anti-RAMP1, -RAMP2, or -RAMP3 antibody (green). Overlapping expression is represented by orange/yellow colour (**C**,**D**,**G**,**H**,**K**,**L**). Blue colour represents 4′,6-diamidino-2-phenylindole (DAPI)-stained DNA. Arrows: Neuroendocrine cells. (**D**,**H**,**L**) represent enlarged sections of (**C**,**G**,**K**). Scale bar: 100 µm (**A**–**C**,**E**–**G**,**I**–**K**), 50 µm (**D**,**H**,**L**).

Similar to the results of the single-labelling experiments, the duodenal epithelium exhibited CALCRL expression in the double-labelling experiments, with particularly intense staining of neuroendocrine cells (Figure 7A,E,I; white arrows). Individual cells in the connective tissue, presumably immune cells, also showed strong CALCRL expression (Figure 7A,E,I). Of the three RAMP isoforms, RAMP2 and RAMP3 were predominantly found in the duodenal epithelium, whereas RAMP1 expression was minimal (Figure 7B,F,J). Accordingly, CALCRL was predominantly co-expressed with RAMP2 and RAMP3 in the duodenal epithelium, with partial co-expression observed in neuroendocrine cells (Figure 7C,D,G,H,K,L; white arrows).

**Figure 8:**
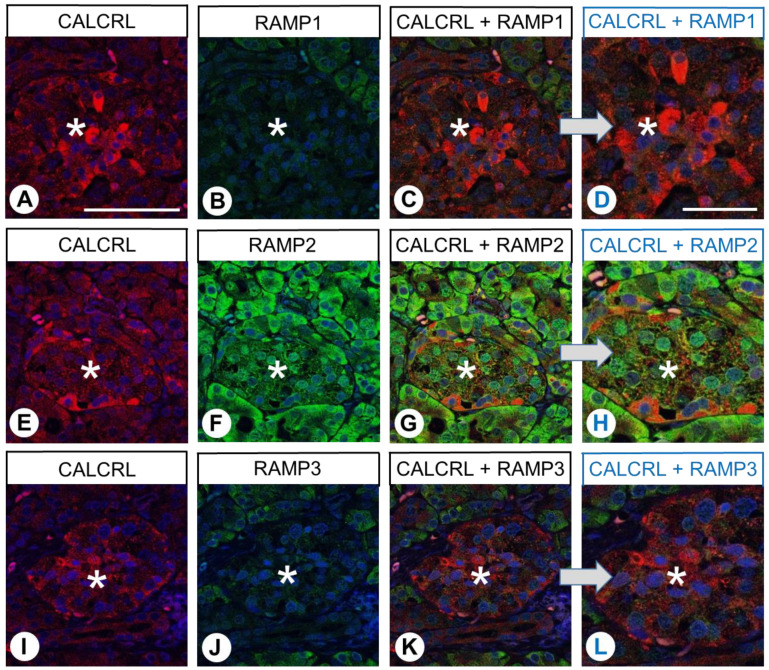
Double-labelling immunohistochemical analysis of calcitonin receptor-like receptor (CALCRL) expression (**A**,**E**,**I**) and the expression of receptor activity-modifying protein (RAMP) 1 (**B**), RAMP2 (**F**), or RAMP3 (**J**) in human pancreatic tissue. Labelling of CALCRL was visualised using Cy3-conjugated anti-rabbit antibody (red). Labelling of RAMP1, RAMP2, or RAMP3 was visualised using Alexa Fluor 488-conjugated rabbit anti-RAMP1, -RAMP2, or -RAMP3 antibody (green). Overlapping expression is represented by orange/yellow colour (**C**,**D**,**G**,**H**,**K**,**L**). Asterisks: Pancreatic islets. Blue colour represents 4′,6-diamidino-2-phenylindole (DAPI)-stained DNA. (**D**,**H**,**L**) represent enlarged sections of (**C**,**G**,**K**). Scale bar: 100 µm (**A**–**C**,**E**–**G**,**I**–**K**), 50 µm (**D**,**H**,**L**).

CALCRL positivity was also evident both in the exocrine and endocrine parts of the pancreas specimens, particularly in a specific peripherally located cell population of the pancreas islets (Figure 8A,E,I). Whereas both exocrine and endocrine pancreatic tissue showed clear expression of RAMP2 (Figure 8F), the extent of RAMP1 and RAMP3 expression was minimal (Figure 8B,J). CALCRL demonstrated some co-expression with RAMP2 in most islet cells, along with partial co-expression in exocrine pancreatic tissue (Figure 8G,H). No such co-expression was observed with RAMP1 or RAMP3 (Figure 8C,D,K,L).

**Figure 9:**
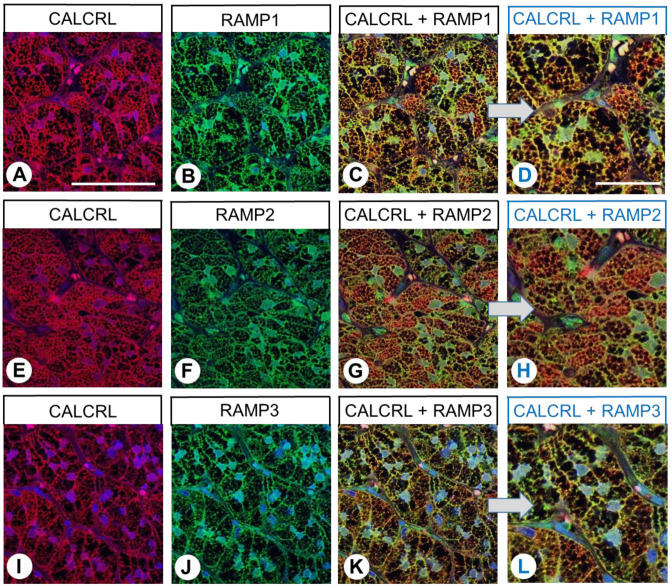
Double-labelling immunohistochemical analysis of calcitonin receptor-like receptor (CALCRL) expression (**A**,**E**,**I**) and the expression of receptor activity-modifying protein (RAMP) 1 (**B**), RAMP2 (**F**), or RAMP3 (**J**) in human adrenocortical tissue. Labelling of CALCRL was visualised using Cy3-conjugated anti-rabbit antibody (red). Labelling of RAMP1, RAMP2, or RAMP3 was visualised using Alexa Fluor 488-conjugated rabbit anti-RAMP1, -RAMP2, or -RAMP3 antibody (green). Overlapping expression is represented by orange/yellow colour (**C**,**D**,**G**,**H**,**K**,**L**). Blue colour represents 4′,6-diamidino-2-phenylindole (DAPI)-stained DNA. (**D**,**H**,**L**) represent enlarged sections of (**C**,**G**,**K**). Scale bar: 100 µm (**A**–**C**,**E**–**G**,**I**–**K**), 50 µm (**D**,**H**,**L**).

In the adrenal cortex, strong CALCRL immunostaining was observed in all three layers (Figure 9A,E,I). All three RAMP isoforms were also clearly expressed (Figure 9B,F,J). Accordingly, CALCRL was co-expressed with RAMP1, RAMP2, and RAMP3 (Figure 9C,D,G,H,K,L).

**Figure 10:**
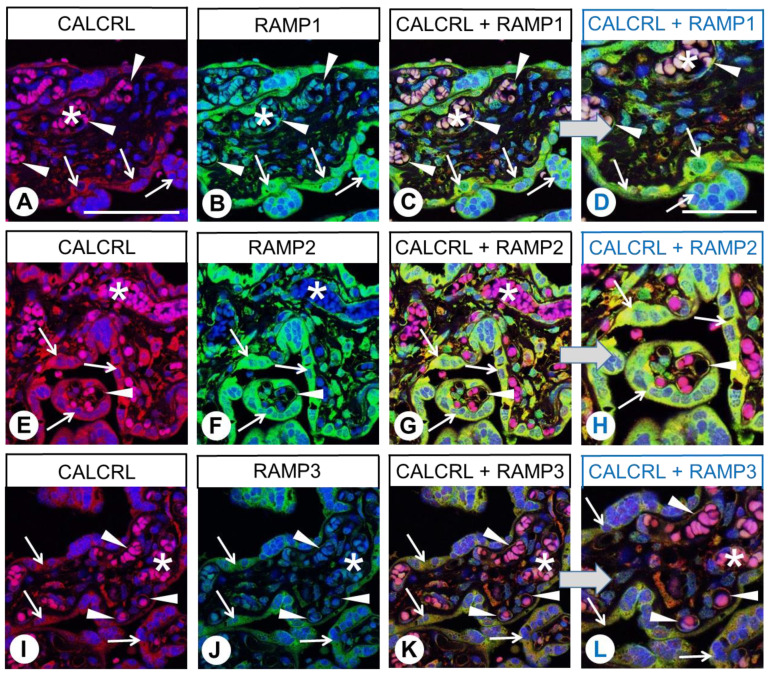
Double-labelling immunohistochemical analysis of calcitonin receptor-like receptor (CALCRL) expression (**A**,**E**,**I**) and the expression of receptor activity-modifying protein (RAMP) 1 (**B**), RAMP2 (**F**), or RAMP3 (**J**) in human placental tissue. Labelling of CALCRL was visualised using Cy3-conjugated anti-rabbit antibody (red). Labelling of RAMP1, RAMP2, or RAMP3 was visualised using Alexa Fluor 488-conjugated rabbit anti-RAMP1, -RAMP2, or -RAMP3 antibody (green). Overlapping expression is represented by orange/yellow colour (**C**,**D**,**G**,**H**,**K**,**L**). Blue colour represents 4′,6-diamidino-2-phenylindole (DAPI)-stained DNA. (**D**,**H**,L) represent enlarged sections of (**C**,**G**,**K**). Arrows: Syncytiotrophoblast cells; arrowheads: Capillary endothelia; asterisks: Non-specifically stained erythrocytes. Scale bar: 100 µm (**A**–**C**,**E**–**G**,**I**–**K**), 50 µm (**D**,**H**,**L**).

In the placenta, CALCRL immunofluorescence was predominantly present in the vascular endothelial cells within the placental villi and in the syncytiotrophoblasts (Figure 10A,E,I; marked by arrowheads and arrows, respectively), whereas, apart from CALCRL-positive immune cells, the connective tissue was negative. RAMP2 expression was also particularly strong in the immune cells, endothelial cells, and syncytiotrophoblasts, whereas the immune signals for RAMP1 and RAMP3 were less prominent overall (Figure 10B,F,J). However, all three RAMP forms exhibited co-expression with CALCRL (Figure 10C,D,G,H,K,L). In addition to these findings, non-specific staining of the erythrocytes (pink staining in the overlay) was also noticeable in the placenta specimens (asterisks).

### 2.3. Immunohistochemical Detection of CALCRL Expression in Various Human Tumour Entities

**Figure 11:**
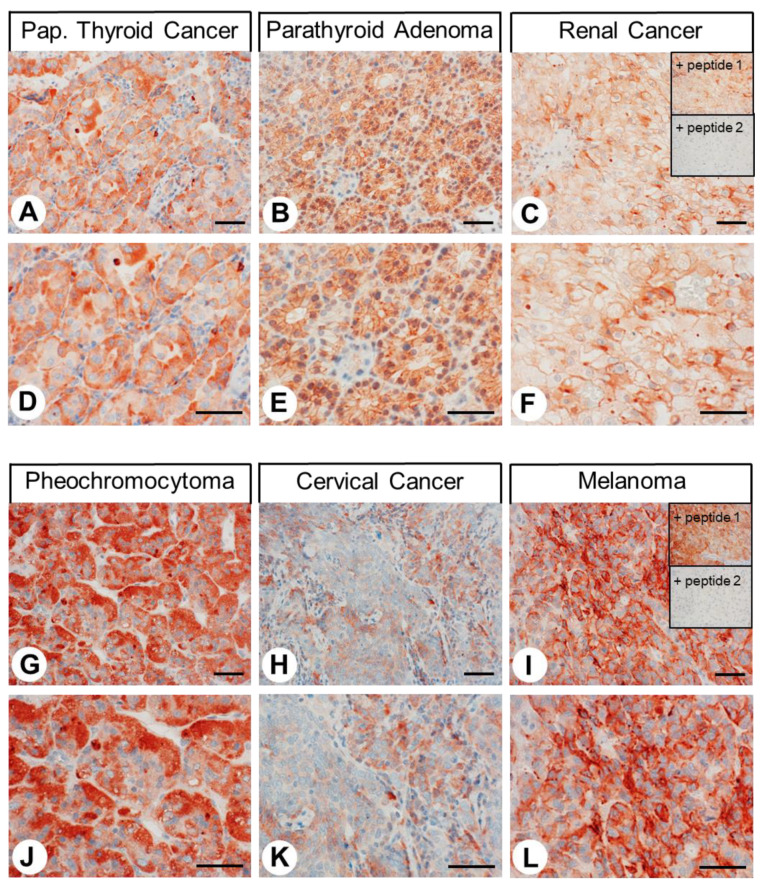
Immunohistochemical detection of calcitonin receptor-like receptor (CALCRL) localisation in human tumour entities. Immunohistochemical staining (red-brown colour) and counterstaining with haematoxylin. (**D**,**E**,**F**) and (**J**,**K**,L) represent enlarged sections of (**A**,**B**,**C**) and (**G**,**H**,**I**). Insets in C and I represent controls for adsorption, in which the anti-CALCRL antibody 8H9L8 was preincubated for 2 h with either 10 µg/mL of a control peptide (peptide 1) or 10 µg/mL of the immunising peptide (peptide 2). Scale bar, 50 µm.

The patterns of CALCRL expression observed in the 32 different tumour entities examined with the corresponding case numbers, the number of CALCRL-positive tumours, and the mean, minimum, and maximum Immunoreactivity Score (IRS) values are summarised in Table 1 (for the calculation of the IRS values from the percentage of stained tumour cells and the intensity of staining, see the “Materials and Methods” section). Higher levels of CALCRL expression, including a greater number of CALCRL-positive cases (IRS ≥ 3) and higher IRS values, were particularly prevalent in all types of thyroid carcinomas, parathyroid adenomas, small-cell lung cancers, large cell neuroendocrine carcinomas of the lung, pancreatic neuroendocrine neoplasms, renal clear cell carcinomas, pheochromocytomas, lymphomas, and melanomas (Table 1). Representative examples of positively stained tumours of these entities are shown in Figure 11 (papillary thyroid cancer, parathyroid adenoma, renal clear-cell cancer, pheochromocytoma, cervical cancer, and melanoma, respectively). A set of normal and neoplastic tissues with positive CALCRL staining was also subjected to immunoadsorption experiments using both a control peptide (peptide 1) and the immunising peptide (peptide 2). Whereas the immunostaining pattern remained unchanged after preincubation with the control peptide, no immunostaining was present after preincubation with the immunising peptide (see insets in Figure 11C,I). Similar to the findings in normal tissues, both cytoplasmic and membranous staining was detected in tumours. As indicated by the minimum and maximum IRS values assigned to individual tumours within each of the different tumour entities and by the respective standard deviations (Table 1), CALCRL expression exhibited substantial interindividual variability. Additionally, as can be seen in Figure 11H,K, in some cases, strong heterogeneity in CALCRL expression between the individual cells of a tumour was noticed.

**Figure 12:**
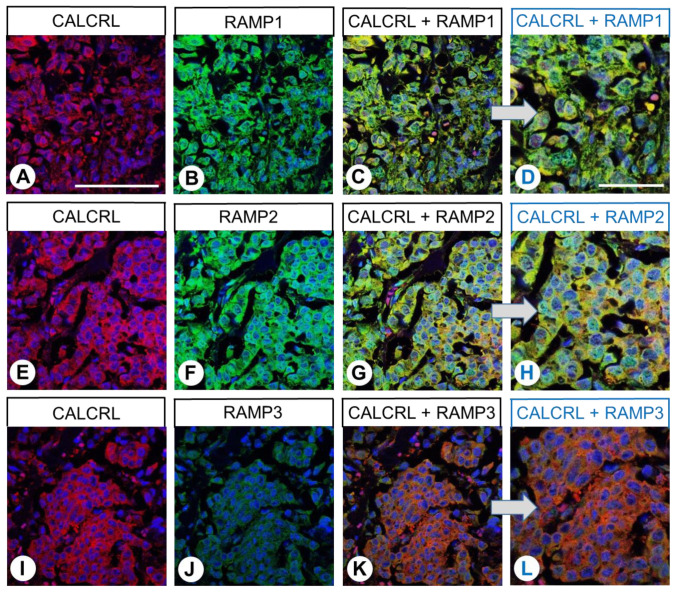
Double-labelling immunohistochemical analysis of calcitonin receptor-like receptor (CALCRL) expression (**A**,**E**,**I**) and the expression of receptor activity-modifying protein (RAMP) 1 (**B**), RAMP2 (**F**), or RAMP3 (**J**) in medullary thyroid cancer tissue. Labelling of CALCRL was visualised using Cy3-conjugated anti-rabbit antibody (red). Labelling of RAMP1, RAMP2, or RAMP3 was visualised using Alexa Fluor 488-conjugated rabbit anti-RAMP1, -RAMP2, or -RAMP3 antibody (green). Overlapping expression is represented by orange/yellow colour. Blue colour represents 4′,6-diamidino-2-phenylindole (DAPI)-stained DNA. (**D**,**H**,**L**) represent (**C**,**D**,**G**,**H**,**K**,**L**) enlarged sections of (**C**,**G**,**K**). Scale bar: 100 µm (**A**–**C**,**E**–**G**,**I**–**K**), 50 µm (**D**,**H**,**L**).

**Figure 13:**
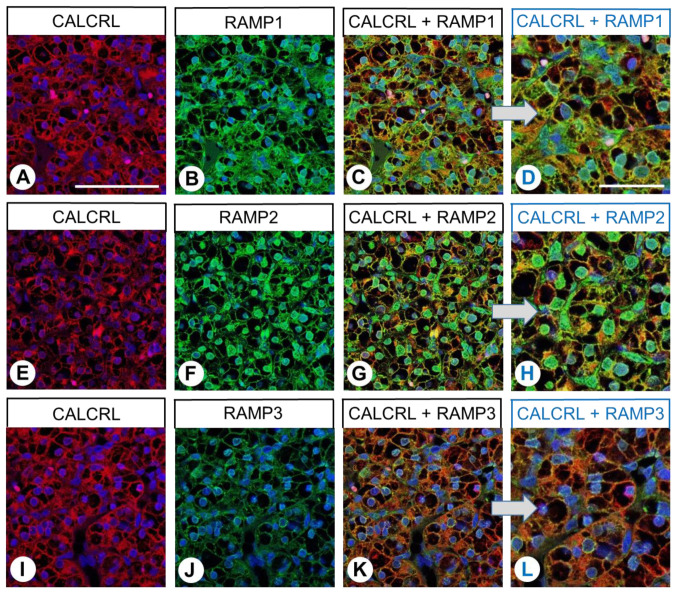
Double-labelling immunohistochemical analysis of calcitonin receptor-like receptor (CALCRL) expression (**A**,**E**,**I**) and the expression of receptor activity-modifying protein (RAMP) 1 (**B**), RAMP2 (**F**), or RAMP3 (**J**) in renal clear cell cancer tissue. Labelling of CALCRL was visualised using Cy3-conjugated anti-rabbit antibody (red). Labelling of RAMP1, RAMP2, or RAMP3 was visualised using Alexa Fluor 488-conjugated rabbit anti-RAMP1, -RAMP2, or -RAMP3 antibody (green). Overlapping expression is represented by orange/yellow colour (**C**,**D**,**G**,**H**,**K**,**L**). Blue colour represents 4′,6-diamidino-2-phenylindole (DAPI)-stained DNA. (**D**,**H**,**L**) represent enlarged sections of (**C**,**G**,**K**). Scale bar: 100 µm (**A**–**C**,**E**–**G**,**I**–**K**), 50 µm (**D**,**H**,**L**).

Subsequently, immunofluorescence double-labelling experiments were performed in medullary thyroid carcinomas, papillary thyroid carcinomas, adenocarcinomas of the lung, renal clear cell carcinomas, pheochromocytomas, lymphomas, and melanomas. These analyses revealed strong expression of CALCRL, robust expression of RAMP2, and moderate expression of RAMP1. With the exception of parathyroid adenomas and melanomas, the tumour specimens in all cases exhibited only low expression of RAMP3. Accordingly, co-expression of CALCRL with all three RAMP isoforms was detected in all examined tumour entities. Some examples of these staining patterns are depicted in Figure 12 and Figure 13 (medullary thyroid cancer, renal clear cell cancer) and in Supplemental Figures S1–S6 (papillary thyroid carcinoma, parathyroid adenoma, adenocarcinoma of the lung, pheochromocytoma, lymphoma, and malignant melanoma, respectively).

## 3. Discussion

### 3.1. Evaluation of the Specificity of the Rabbit Anti-Human CALCRL Antibody

Monoclonal antibodies have the advantage over polyclonal ones that they are directed against a single epitope only, thus leading to more specific staining results. Additionally, they are available in unlimited quantities over an unlimited period of time and always with the same quality. Here, we developed a rabbit monoclonal anti-CALCRL antibody that could be used for immunohistochemical analyses of formalin-fixed, paraffin-embedded human tissues during routine histopathology procedures. We showed that the antibody is additionally well suited for immunocytochemistry experiments and Western blot analyses and for the detection of CALCRL at the protein level in rat and mouse tissues in basic research. In the present study, we demonstrated that the novel rabbit anti-CALCRL antibody 8H9L8 specifically detects its targeted receptor and does not cross-react with other proteins. First, in Western blot analyses using membrane preparations from BON-1 cells that endogenously express the receptor, the anti-CALCRL antibody detected a band at approximately 66 kDa, which is consistent with the expected molecular weight of the glycosylated receptor [33,34]. Second, the immunoreactive band was completely extinguished after the antibody was preadsorbed with the immunising peptide, but the band remained visible after the antibody was incubated with the control peptide that corresponded to a different region of the receptor. Furthermore, CALCRL knockdown with a CALCRL-specific siRNA led to the complete absence of the immunoreactive band.

In the immunocytochemistry experiments, the antibody demonstrated both membranous and cytoplasmic staining of BON-1 cells. The immunostaining intensity was substantially reduced by treatment of the cells with a CALCRL-specific siRNA. Finally, immunostaining was completely absent in BON-1 cells and CALCRL-positive tissue specimens after the antibody was preadsorbed with its immunising peptide, but immunostaining was unaffected by preadsorption with the control peptide.

Furthermore, although immunocytochemical double-labelling experiments in BON-1 cells primarily revealed the presence of RAMP1 and RAMP2, expression of RAMP3 could also be observed, and all three proteins clearly co-expressed with CALCRL, suggesting the presence of CGRPR, the AM_1_ receptor, and the AM_2_ receptor in these cells.

### 3.2. Immunohistochemical Detection of CALCRL Expression in Normal Human Tissues

The detection of a consistent CALCRL expression in **capillary** endothelia, as well as smooth muscles in the **arterioles and arteries** in the tissue samples investigated, is consistent with published data concerning the expression of CALCRL and the co-expression of CALCRL/RAMP1, as well as the distribution of the peptides CGRP, adrenomedullin, and amylin, all three of which are considered potent vasodilators [4,35,36,37,38,39,40,41]. In many normal and neoplastic tissues, CALCRL was found on **immune cells**, which were generally presumed to comprise monocytes and macrophages. These findings are consistent with the reported immunomodulatory effects of both CGRP and adrenomedullin [4,6,42,43,44,45], as well as the presence of adrenomedullin and its receptors in numerous immune cells (e.g., macrophages, monocytes, T cells, and dendritic cells) [43].

In human, rat, and mouse cerebral **cortex** specimens, CALCRL was detected in distinct cell populations, which presumably represent both pyramidal and glial cells. In previous studies, the ligands CGRP and adrenomedullin have been observed in or near the vasculature, in addition to widespread expression in the neuronal tissues of various brain regions, where they modulate processes such as the activities of various autonomic centres involved in water and electrolyte balance and food intake [1,4,6,7]. Additionally, GRCP and adrenomedullin have modulating influences on the synthesis and release of neuropeptides, such as somatotropin, luteinising hormone, oxytocin, and prolactin [46]. Notably, CGRP-containing neurons and nerve fibres have been detected in brain regions such as the thalamus, hypothalamus, midbrain, brainstem, and hippocampus but not in the cerebral cortex. Adrenomedullin has also been found in the cerebral cortex, where it was detected in the pyramidal cells of layers I to VI [46]. In situ hybridisation analyses of rat brain tissue showed that RAMP1 is primarily expressed in the cerebral cortex, caudate–putamen, and olfactory tubercle; RAMP2 is predominantly expressed in the hypothalamus; and RAMP3 is primarily expressed in thalamic nuclei [47,48,49]. Our finding of CALCRL expression on cortical pyramidal cells is most similar to the pattern of RAMP1 (and hence CGRPR) expression.

In all three species investigated, CALCRL was detectable both in the **adeno- and in the neurohypophysis**. Our CALCRL/RAMP immunofluorescence double-labelling experiments showed that the expression of the AM_1_ receptor (co-expression of CALCRL and RAMP2) was predominant in the human anterior pituitary gland; the AMY_1_ receptor and the AMY_3_ receptor were also expressed in some other cell populations (i.e., CALCRL was absent, but RAMP1 or RAMP3 was present). The predominance of AM_1_ receptor expression observed in this study is consistent with previous findings, whereby adrenomedullin binding sites were primarily detected in the adenohypophysis and the neurohypophysis. Adrenomedullin in the adenohypophysis reportedly leads to decreased adrenocorticotropic hormone and increased growth hormone release [46,50,51]. Although no amylin expression in the pituitary gland has been reported in the literature [52], amylin production at other sites (e.g., the pancreas) inhibits prolactin release in the pituitary gland [53].

Strong CALCRL immunostaining was also observed in the **dorsal root ganglia** of all three species investigated. The reported expression of CTR, CALCRL, and all three RAMP isoforms in the trigeminal and dorsal root ganglia suggest the presence and involvement of CGRPR and several other members of the CTR family in nociceptive transmission [16,17,18,20,21,23,49,54].

The finding of a moderate CALCRL expression in the smooth muscles, bronchial epithelium, and bronchial glands of **larger bronchi** is consistent with existing literature, in which ^125^I-CGRP binding sites were detected in the muscle layer of the bronchial and pulmonary vasculature, the bronchial epithelium, the bronchial musculature, and the bronchial glands of various species (including humans) [55]. The localisation of such CGRP binding sites in the bronchial musculature is compatible with the known constrictive effects of the peptide on the smooth muscle cells in all airway compartments in humans [56]. The prominent expression of CGRP binding sites in the bronchial and pulmonary vasculature is consistent with the presence of CGRP-immunoreactive nerve fibres in the vascular environment [57,58].

The analysis of **pancreatic** tissue specimens from all three species investigated revealed particularly intense staining in a very small subpopulation of islet cells (i.e., these cells were not present in some pancreatic islet sections). In terms of location and number, these cells may correspond to somatostatin-producing δ-cells or to cells that secrete pancreatic polypeptide. Because these islet cells and β-cells exhibited co-expression of CALCRL with RAMP2, these islet cells presumably exhibit predominant expression of AM_1_ receptor, whereas the outer α-cells and the exocrine pancreas show expression of AMY_2_ receptor (low CALCRL but substantial RAMP2 expression). Amylin is produced in the β-cells of the pancreas and is released along with insulin after a meal. It inhibits glucagon secretion from the α-cells of the pancreas, thereby reducing the release of glucose from the liver through the suppression of glycogenolysis and gluconeogenesis [5,59]. Thus, AMY_2_ receptor expression by the α-cells of the pancreas was expected. Amylin also causes delayed gastric emptying and centrally mediates an increased feeling of satiety [59]. Amylin and its analogues, such as pramlintide, are, therefore, regarded as potential treatments for diabetes mellitus and obesity [13,59,60,61,62,63]. By contrast, the secretion of adrenomedullin from pancreatic polypeptide-producing cells leads to decreased insulin release from pancreatic β-cells [50,64,65]. Accordingly, our finding of AM_1_ receptor expression by β-cells was also expected and was consistent with published data [50]. In contrast to the endocrine pancreas, the expression of calcitonin family receptors in the exocrine pancreas has not been extensively studied thus far. Previous studies in guinea pigs revealed ^125^I-CGRP binding sites in individual pancreatic acini [66,67], but these receptors have not been extensively characterised. There have also been contradictory reports regarding the effects that might be mediated by these receptors. Whereas CGRP caused increased amylase secretion in isolated guinea pig or rat acinar cells [66,67,68], it caused a reduction in pancreatic secretion in experimental rats [68,69].

In human, rat, and mouse **kidney** specimens, CALCRL expression was only detected in the vasculature. Notably, the literature contains evidence only concerning the influences of CGRP and adrenomedullin on the renal vascular system [70,71,72,73].

Both **adrenal** cortex and adrenal medulla specimens of all three species exhibited CALCRL expression. Double-labelling experiments involving the human adrenal cortex revealed co-expression of CALCRL with all three RAMP isoforms, which indicated the presence of CGRPR, the AM_1_ receptor, and the AM_2_ receptor. In previous studies, CGRPR and adrenomedullin binding sites have been found throughout the adrenals, and adrenomedullin has demonstrated an ability to increase catecholamine release from the adrenal medulla [6,50,74,75,76]. Regarding the effect of adrenomedullin on aldosterone secretion from the zona glomerulosa of the adrenal cortex, the available data are contradictory; some studies have demonstrated the inhibition of aldosterone release [6,50,77,78,79], whereas other studies have shown stimulation [50,80].

In this study, all normal tissue specimens from the **gastrointestinal tract** showed substantial CALCRL expression in the mucosa, particularly among enteroendocrine cells (which were identified based on their number, morphology, and localization in the intestinal epithelium) and infiltrating immune cells. In particular, CALCRL colocalisation with RAMP3 and (to a lesser extent) with RAMP1 in the mucosa indicates the expression of the AM_2_ receptor and CGRPR. Sensory CGRP-containing nerve fibres permeate all layers of the entire gastrointestinal tract [81], and CGRP is involved in the regulation of gastrointestinal blood flow, in addition to numerous other effects, such as inhibiting the secretion of gastric acid, reducing the motility of the gastrointestinal tract, and modulating visceral nociception [82,83]. In previous studies, adrenomedullin expression has been observed in the enteroendocrine cells of the gastrointestinal mucosa, the chief cells of the gastric fundus, and the submucosa of the duodenum, ileum, and colon, whereas expression of the corresponding receptor components was detected in the enteric ganglia [84,85]. Overall, adrenomedullin is presumed to function in a manner similar to CGRP and to participate in maintaining the homeostasis of the gastrointestinal tract.

In the human **placenta**, in particular co-expression patterns with RAMP2 and RAMP1 were observed, indicating the expression of AM_1_ receptor and CGRPR. These results are consistent with published data, which show that CALCRL and RAMP1 are expressed in the endothelium and in the muscle layer of the umbilical, chorionic, and villus vasculature, as well as in trophoblasts in the human [86,87]. Furthermore, CGRP reportedly has an important role in trophoblast morphological and functional differentiation [88], as well as physiological maternal hemodynamic adaptation and foetal growth through dilatation of the umbilical cord and placental vasculature, which leads to improved fetoplacental perfusion [87]. The presence of adrenomedullin and amylin has also been detected in the human placenta, and amylin is particularly detectable in syncytiotrophoblasts in early pregnancy [89,90]. Similar to CGRP, adrenomedullin relaxes the placental vasculature [89].

### 3.3. Immunohistochemical Detection of CALCRL Expression in Human Neoplastic Tissues

Among the 32 tumour entities investigated, strong CALCRL expression was present in all types of thyroid carcinomas, as well as parathyroid adenomas, small-cell lung cancers, large cell neuroendocrine carcinomas of the lung, pancreatic neuroendocrine neoplasms, renal clear cell carcinomas, pheochromocytomas, lymphomas, and melanomas. Additionally, single cases with strong CALCRL expression were observed in many of the other tumour entities. Subsequent immunofluorescence double-labelling experiments performed on various CALCRL-positive tumour specimens revealed that RAMP2 expression was predominant, with slightly lower expression of RAMP1. With the exception of melanomas, the tumour specimens exhibited minimal expression of RAMP3. These findings suggest that the AM_1_ receptor and CGRPR are the main calcitonin family receptors in tumours, whereas the AM_2_ receptor exhibits lower expression.

The expression of adrenomedullin in neoplastic tissues has received increasing attention in recent years. In tumours, such as colorectal carcinomas [91], pancreatic carcinomas [92,93,94,95], gastroenteropancreatic neuroendocrine neoplasms [96,97], hepatocellular carcinomas [98], renal cancer [99,100], pheochromocytomas [101], prostate cancer [102,103,104,105,106], breast cancer [107,108,109], ovarian cancer [110,111,112,113], endometrial cancer [114,115], cervical cancer [116,117], and malignant melanomas [118], increased adrenomedullin levels have been linked to a reduced anti-tumour immune response, increased neoangiogenesis, decreased tumour cell apoptosis, enhanced tumour proliferation, stronger metastasis, and worse patient prognosis.

Compared with adrenomedullin, there is considerably less information available concerning the expression patterns and functions of CGRP in tumours. CGRP has proangiogenic and prolymphangiogenic properties; thus, it can enhance tumour-associated angiogenesis and tumour growth [119]. CGRP expression has been detected in a high percentage of medullary thyroid carcinomas [120,121], in small-cell lung carcinomas and corresponding cell lines [122,123], and in hepatocellular carcinomas and corresponding cell lines [124]. Notably, CGRP can increase the invasive and migratory capacities of cultured prostate cancer cells by 30–40%, and there is some evidence that CGRP (via CGRPR) promotes prostate cancer metastasis to bone [106].

In contrast to adrenomedullin (and CGRP), minimal information is available concerning the expression patterns of CALCRL and the various RAMP isoforms in human tumours. Expression patterns have been reported for human glioblastomas and glioblastoma cell lines (CALCRL, RAMP1/2/3) [125,126,127], gastric cancer (CALCRL, RAMP1/2/3) [128], colorectal cancer (CALCRL, RAMP2/3) [91], pancreatic cancer (CALCRL, RAMP1/2/3) [93,94], hepatocellular carcinomas and corresponding cell lines (CALCRL, RAMP1/2/3) [98,124], renal cancer (CALCRL, RAMP2/3) [100], pheochromocytomas (CALCRL, RAMP2) [129], prostate cancer and corresponding cell lines (CALCRL, RAMP2/3) [103,104], breast cancer and corresponding cell lines (CALCRL, RAMP2/3) [109], and ovarian cancer (CALCRL) [111]. However, most studies thus far have been limited to analyses of mRNA.

To our knowledge, the present investigation included the first examination of CALCRL expression in 21 of the 32 tumour entities analysed. These tumour types were papillary, follicular, medullary, and anaplastic thyroid carcinomas; parathyroid adenomas; squamous cell carcinomas; adenocarcinomas and large cell neuroendocrine carcinomas of the lung; small-cell lung cancer; gastrointestinal stromal tumours; intestinal and pancreatic neuroendocrine neoplasms; cholangiocellular carcinomas; renal clear cell carcinomas; testicular, endometrial, and cervical cancers; lymphomas; leiomyosarcomas; rhabdomyosarcomas; and liposarcomas. We found that thyroid carcinomas, parathyroid adenomas, pancreatic neuroendocrine tumours, renal clear cell carcinomas, and lymphomas exhibited strong CALCRL expression in a high percentage of specimens. Because such expression may have therapeutic relevance, further investigations with larger numbers of cases should be conducted in these and other tumour entities with strong expression of CALCRL.

## 4. Materials and Methods

### 4.1. Antibody

Through a collaboration with Thermo Fisher Scientific (Waltham, MA, USA), a rabbit monoclonal antibody, 8H9L8, was produced against an amino acid sequence in the N-terminal region of human CALCRL. This antibody can also be obtained from Thermo Fisher Scientific (Catalog no.: 703811), and to the best of our knowledge, 8H9L8 is the only rabbit monoclonal anti-CALCRL antibody commercially available so far. The peptide used to immunise the rabbits (i.e., the immunising peptide) was CYQKIMQDPIQQAEGVY, which corresponds to residues 48–64 of human CALCRL. The corresponding mouse CALCRL sequence is CYQKIMQDPIQQAEG**L**Y, and that of the rat reads CYQKIMQDPIQQ**G**EG**L**Y. Although these sequences differ by 1–2 amino acids from the sequence of human CALCRL, the monoclonal antibody 8H9L8 cross-reacts with the mouse and rat forms of the receptor.

### 4.2. Western Blot Analysis

Endogenous CALCRL-expressing BON-1 cells (DSMZ, Braunschweig, Germany) were seeded onto poly-L-lysine-coated 60-mm dishes and grown to 80% confluence. Cells were either left untreated or treated with chemically synthesised, double-stranded CALCRL siRNA duplexes (Santa Cruz Biotechnology, Dallas, TX, USA) in accordance with the manufacturer’s instructions. A scrambled siRNA was used as the negative control (Santa Cruz Biotechnology). Subsequently, the cells were lysed in detergent buffer (20 mM 4-(2-hydroxyethyl)-1-piperazineethanesulfonic acid [HEPES, pH 7.4], 150 mM NaCl, 5 mM ethylenediaminetetraacetic acid, 1% Triton X-100, 10% glycerol, 0.1% sodium dodecyl sulphate, 0.2 mM phenylmethylsulfonylfluoride, 10 mg/mL leupeptin, 1 mg/mL pepstatin A, 1 mg/mL aprotinin, and 10 mg/mL bacitracin). CALCRL enrichment was conducted using wheat germ lectin agarose beads (J-OIL MILLS, Inc., Tokyo, Japan), as previously described [130]. Subsequently, the protein content of the samples was determined using the Pierce™ BCA Protein Assay Kit (Thermo Fisher Scientific, Waltham, MA, USA) according to the manufacturer’s instructions, and the samples (20 µg of protein per lane) were subjected to 7.5% sodium dodecyl sulphate-polyacrylamide gel electrophoresis and immunoblotted onto polyvinylidene fluoride membranes. Blots were incubated with the rabbit monoclonal anti-CALCRL antibody 8H9L8 (1:500 dilution) overnight at 4 °C, then incubated with peroxidase-conjugated secondary anti-rabbit antibody (1:5000 dilution; Santa Cruz Biotechnology) for 2 h at room temperature and visualised by enhanced chemiluminescence (Amersham, Braunschweig, Germany).

For adsorption controls, the anti-CALCRL antibody was preincubated for 2 h at room temperature with either 10 µg/mL of the immunising peptide (peptide 2) or 10 µg/mL of a control peptide that corresponded to a different region of the receptor (peptide 1; residues 23–40; sequence: ELEESPEDSIQLGVTRNK).

### 4.3. Immunocytochemistry

Endogenous CALCRL-expressing BON-1 cells (DSMZ) were seeded onto coverslips and grown to 80% confluence. The cells were then either left untreated or treated with chemically synthesised, double-stranded CALCRL siRNA duplexes (Santa Cruz Biotechnology) in accordance with the manufacturer’s instructions. A scrambled siRNA was used as the negative control (Santa Cruz Biotechnology). Next, the cells were washed with phosphate-buffered saline and fixed with 4% paraformaldehyde and 0.2% picric acid in phosphate buffer (pH 6.9) for 20 min at room temperature. After the fixed cells had been thoroughly washed with phosphate-buffered saline, they were incubated with the anti-CALCRL antibody 8H9L8 (1:500 dilution) overnight at 4 °C, then incubated with the Alexa Fluor 488-conjugated anti-rabbit secondary antibody (1:5000 dilution; Invitrogen, Carlsbad, CA, USA) for 2 h at room temperature. Finally, the fixed cells were mounted using Fluoromount G (Invitrogen) and analysed using an LSM 510 META laser scanning confocal microscope (Carl Zeiss, Jena, Germany). To establish controls for immunostaining analyses, the anti-CALCRL antibody was either omitted or adsorbed for 2 h at room temperature with 10 µg/mL of the immunising peptide (peptide 2) or with 10 µg/mL of a control peptide that corresponded to a different region of the receptor (peptide 1, described above).

Double-labelling immunocytochemistry with CALCRL and RAMP1, RAMP2, or RAMP3 was conducted by first incubating fixed cells with the anti-CALCRL antibody 8H9L8 (1:500) overnight at 4 °C. On the following day, the cells were washed with phosphate-buffered saline and incubated for 2 h in darkness at room temperature with Cy3-conjugated anti-rabbit secondary antibody (1:1000 dilution; Dianova, Hamburg, Germany). The cells were then washed thoroughly with phosphate-buffered saline and incubated overnight at 4 °C with Alexa 488-conjugated rabbit polyclonal anti-RAMP1, RAMP2, or RAMP3 antibody (1:100 dilution; Bioss Antibodies, Woburn, MA, USA; catalogue numbers, bs-1567R-A488; bs-11971R-A488; bs-11972R-A488; the antibodies were tested beforehand for specificity by means of siRNA knockdown experiments in BON-1 cells, which (as could also be shown in the present paper) endogenously express all three RAMP proteins [siRNAs used: RAMP1, sc-40894; RAMP2, sc-3678; RAMP3, sc-40896; Santa Cruz Biotechnology; Supplemental Figure S7] and by means of peptide neutralisations in RAMP-positive tissues (duodenum; placenta); Supplemental Figure S8). Finally, the cells were coverslipped (Fluoromount G, with 4′,6-diamidino-2-phenylindole [DAPI]; Thermo Fisher Scientific) and analysed using an LSM 510 META laser scanning confocal microscope (Carl Zeiss; magnification: 400×; excitation wavelengths: 405 nm (DAPI); 488 nm (Alexa-488); 543 nm (Cy3)).

### 4.4. Immunohistochemical Evaluation of CALCRL Expression in Normal and Neoplastic Tissues

#### 4.4.1. Tissue Specimens

For the evaluation of CALCRL expression in various human tumour entities, 290 completely anonymised, archived, formalin-fixed, and paraffin-embedded tumour specimens from 290 patients (for the 32 different tumour entities examined, covering the most important human tumours, and the corresponding case numbers, see Table 1) were obtained from the Laboratory of Pathology and Cytology Bad Berka (Bad Berka, Germany). Many of the tumour specimens contained adjacent non-neoplastic tissue, which was also evaluated. Additionally, in order to obtain as complete a CALCRL expression profile as possible for the human body, archived tumour-free human tissue specimens from the cerebral cortex, pituitary, dorsal root ganglia, thymus, spleen, lymph nodes, lung, heart, liver, duodenum, jejunum, ileum, colon, pancreas, kidney, adrenals, and testicles (n = 5–10 each) were obtained from the Laboratory of Pathology and Cytology Bad Berka for this study.

All procedures involving human participants in this study were performed in accordance with the 1964 Declaration of Helsinki and its later amendments. The local ethics committee (Ethikkommission der Landesärztekammer Thüringen) granted permission for this retrospective analysis. For this type of study involving completely anonymised human specimens that had been archived for >10 years, for which clinical data were not obtained, formal patient consent was not required.

For the evaluation of CALCRL expression in rat and mouse tissues, formalin-fixed and paraffin-embedded specimens from normal rat and mouse brain, pituitary, dorsal root ganglia, thymus, spleen, lymph nodes, lung, heart, liver, duodenum, jejunum, ileum, colon, pancreas, kidneys, adrenals, and testicles (n = 6 each; male animals only) were obtained from the archives of the Institute of Pharmacology and Toxicology, Jena University Hospital, Friedrich Schiller University Jena, Jena, Germany.

#### 4.4.2. Immunohistochemistry

From the paraffin-embedded tissue specimens, 4-µm-thick sections were prepared and floated onto positively charged slides. After the sections were dried at room temperature, they were deparaffinised and rehydrated through a graded ethanol series. Endogenous peroxidases were then blocked by incubation in 0.3% H_2_O_2_ in methanol for 45 min. Subsequently, sections were microwaved in 10 mM citric acid (pH 6.0) for 16 min at 600 W.

Single-labelling immunohistochemistry was conducted by an indirect peroxidase labelling method. Sections were first incubated with the anti-CALCRL antibody 8H9L8 (1:500 dilution) overnight at 4 °C, then incubated with biotinylated anti-rabbit IgG and peroxidase-conjugated avidin (Vector ABC “Elite” Kit; Vector Laboratories, Burlingame, CA, USA) for 30 min each at room temperature. The chromogen comprised 3-amino-9-ethylcarbazole in acetate buffer (BioGenex, San Ramon, CA, USA). Sections were counterstained with Mayer’s haematoxylin and mounted in Vectamount™ mounting medium (Vector Laboratories). To establish controls for immunohistochemical analyses, the anti-CALCRL antibody 8H9L8 was either omitted, replaced by a rabbit IgG isotype control (ab125938; 1:100 dilution; Abcam, Cambridge, UK), or adsorbed for 2 h at room temperature with 10 µg/mL of the immunising peptide (peptide 2) or a control peptide that corresponded to a different region of the receptor (peptide 1, described above; see insets in Figure 11C,I)).

Double-labelling fluorescence immunohistochemistry with CALCRL and RAMP1, RAMP2, or RAMP3 was conducted by first incubating the sections with the anti-CALCRL antibody 8H9L8 (1:500 dilution) overnight at 4 °C. The sections were then washed with phosphate-buffered saline/1% bovine serum albumin and incubated for 2.5 h in darkness at room temperature with Cy3-conjugated anti-rabbit secondary antibody (1:1000 dilution; Dianova). After sections had been thoroughly washed with phosphate-buffered saline/1% bovine serum albumin, they were incubated overnight at 4 °C with the Alexa 488-conjugated rabbit polyclonal anti-RAMP1, -RAMP2, or -RAMP3 antibody (1:100 dilution; Bioss Antibodies; catalogue numbers, bs-1567R-A488; bs-11971R-A488; bs-11972R-A488; regarding specificity testing, see above). Finally, sections were mounted (Fluoromount G, with DAPI; Thermo Fisher Scientific) and evaluated using an LSM 510 META laser scanning confocal microscope (Carl Zeiss; magnification: 400×; excitation wavelengths: 405 nm (DAPI); 488 nm (Alexa-488); 543 nm (Cy3)).

The results of CALCRL single-labelling staining with the chromogen 3-amino-9-ethylcarbazole in human tumour specimens were scored using the semiquantitative Immunoreactivity Score (IRS) described by Remmele and Stegner (1987) [131]. The percentage of positive tumour cells in each of the five grades (no positive cells, 0; <10% positive cells, 1; 10–50% positive cells, 2; 51–80% positive cells, 3; and >80% positive cells, 4) was multiplied by the staining intensity assessed in four categories (no staining, 0; mild staining, 1; moderate staining, 2; and strong staining, 3). Thus, IRS values of 0–12 were obtained. Only tumours with an IRS ≥ 3 were regarded as CALCRL-positive. All specimens were evaluated by two independent, blinded investigators (BW, AL). In cases of disagreement, final decisions were made by consensus. Photomicrographs of the single-labelling immunohistochemistry staining were taken with an Axio.Imager A1 microscope (Carl Zeiss) at a magnification of 400× (ocular, 10×/23; objective, 40×/0.75).

## 5. Conclusions

We have generated and characterised a novel rabbit monoclonal anti-human CALCRL antibody that is well suited for visualising CALCRL expression in formalin-fixed, paraffin-embedded human, rat, and mouse tissues. This antibody is also suitable for Western blot analyses and immunocytochemistry experiments. To our knowledge, this antibody has facilitated the establishment of the first broad profile of CALCRL protein expression in diverse normal and neoplastic tissues from humans, as well as normal tissues from rats and mice. In addition to the confirmation of previous findings, this antibody enabled us to provide the first descriptions of CALCRL expression in many tumour entities. Among the tumour types investigated in this study, CALCRL was predominantly expressed in all types of thyroid carcinomas, as well as parathyroid adenomas, small-cell lung cancers, large cell neuroendocrine carcinomas of the lung, pancreatic neuroendocrine neoplasms, renal clear cell carcinomas, pheochromocytomas, lymphomas, and melanomas. In these tumours, CALCRL may represent a useful target structure for future therapies.

## Figures and Tables

**Table 1:** Calcitonin receptor-like receptor (CALCRL) statuses of various tumour entities. Min, Max, Mean: Minimum, maximum, and mean Immunoreactivity Score (IRS) values; SD: Standard deviation. Tumours with a mean IRS ≥ 3.0 are indicated in bold font.

Tumour Type (Number of Cases [n])	CALCRL-Positive Tumours [n] (%)	IRS			
		**Min**	**Max**	**Mean**	**SD**
Glioblastoma (9)	0 (0%)	0	1	0.67	0.50
**Thyroid carcinoma (37)**	**29 (78.4%)**	**0**	**10.5**	**5.22**	**2.71**
**- Papillary (11)**	**8 (72.7%)**	**2**	**10.5**	**5.82**	**3.48**
**- Follicular (9)**	**7 (77.8%)**	**0**	**6**	**4.78**	**2.22**
**- Medullary (9)**	**8 (88.9%)**	**1**	**9**	**5.33**	**2.12**
**- Anaplastic (8)**	**6 (75.0%)**	**2**	**9**	**4.75**	**2.92**
**Parathyroid adenoma (10)**	**10 (100%)**	**4**	**12**	**6.65**	**3.16**
**Lung cancer (40)**	**14 (35.0%)**	**1**	**12**	**3.55**	**2.82**
**- Squamous cell carcinoma (10)**	**6 (60.0%)**	**1**	**8**	**3.95**	**2.57**
- Adenocarcinoma (9)	4 (44.4%)	2	6	2.78	1.48
**- Small cell lung cancer (13)**	**7 (53.8%)**	**0**	**12**	**3.73**	**3.77**
**- Large cell neuroendocrine carcinoma (8)**	**3 (37.5%)**	**1**	**8**	**3.63**	**2.83**
Gastric adenocarcinoma (16)	1 (6.3%)	0	6	1.66	1.42
Colon carcinoma (8)	4 (50.0%)	0	6	2.38	2.00
Gastrointestinal stromal tumour (10)	0 (0%)	0	2	0.50	0.82
Pancreatic adenocarcinoma (8)	4 (50.0%)	0	6	2.81	1.96
Intestinal neuroendocrine neoplasm (12)	3 (25.0%)	0	6	1.42	2.27
**Pancreatic neuroendocrine neoplasm (10)**	**10 (100%)**	**3**	**10**	**6.70**	**1.89**
Hepatocellular carcinoma (10)	1 (10.0%)	0	3	0.70	1.03
Cholangiocellular carcinoma (10)	3 (30.0%)	0	6	2.30	1.89
**Renal clear cell carcinoma (12)**	**10 (83.3%)**	**2**	**9**	**5.46**	**2.25**
Urinary bladder cancer (7)	2 (28.6%)	0	3	1.14	1.35
**Pheochromocytoma (6)**	**6 (100%)**	**6**	**8**	**7.00**	**1.10**
Prostate adenocarcinoma (12)	4 (33.3%)	1	4	2.25	1.22
Testicular cancer (12)	2 (16.7%)	0	6	1.50	1.68
Breast carcinoma (9)	0 (0%)	0	1	0.67	0.50
Endometrial cancer (10)	2 (20.0%)	1	3	2.10	0.57
Cervical cancer (9)	1 (11.1%)	1	4	1.89	0.93
Ovarian cancer (9)	1 (11.1%)	0	3	1.44	0.88
**Lymphoma (10)**	**6 (60.0%)**	**0**	**12**	**4.45**	**4.35**
**Melanoma (5)**	**4 (80.0%)**	**3**	**10**	**6.60**	**2.61**
Leiomyosarcoma (3)	0 (0%)	0	1	0.25	0.50
Rhabdomyosarcoma (3)	0 (0%)	0	0	0	0
Liposarcoma (3)	1 (33.3%)	0	4	1.33	2.31

## Data Availability

The data that support the findings of this study are all contained within the article.

## Supplementary Materials

The following supporting information can be downloaded at: https://www.mdpi.com/article/10.3390/ijms24043960/s1.

## Abbreviations

AM_1_ receptor, adrenomedullin 1 receptor; AM_2_ receptor, adrenomedullin 2 receptor; AMY_1_ receptor, amylin 1 receptor; AMY_2_ receptor, amylin 2 receptor; CALCRL, calcitonin receptor-like receptor; CGRP, calcitonin gene-related peptide; CGRPR, calcitonin gene-related peptide receptor; CTR, calcitonin receptor; IRS, Immunoreactivity Score; RAMP, receptor activity-modifying protein; siRNA, small interfering RNA.
