# Supplementary material for: Expression of the Calcitonin Receptor-like Receptor (CALCRL) in Normal and Neoplastic Tissues
Source: Int J Mol Sci. 2023 Feb 16;24(4):3960. doi: 10.3390/ijms24043960 (PMC9962437; doi:10.3390/ijms24043960)
Supplement: Supplementary file 1 [file ijms-24-03960-s001.zip › ijms-2059537-supplementary.pdf]

Supplemental Figure S1

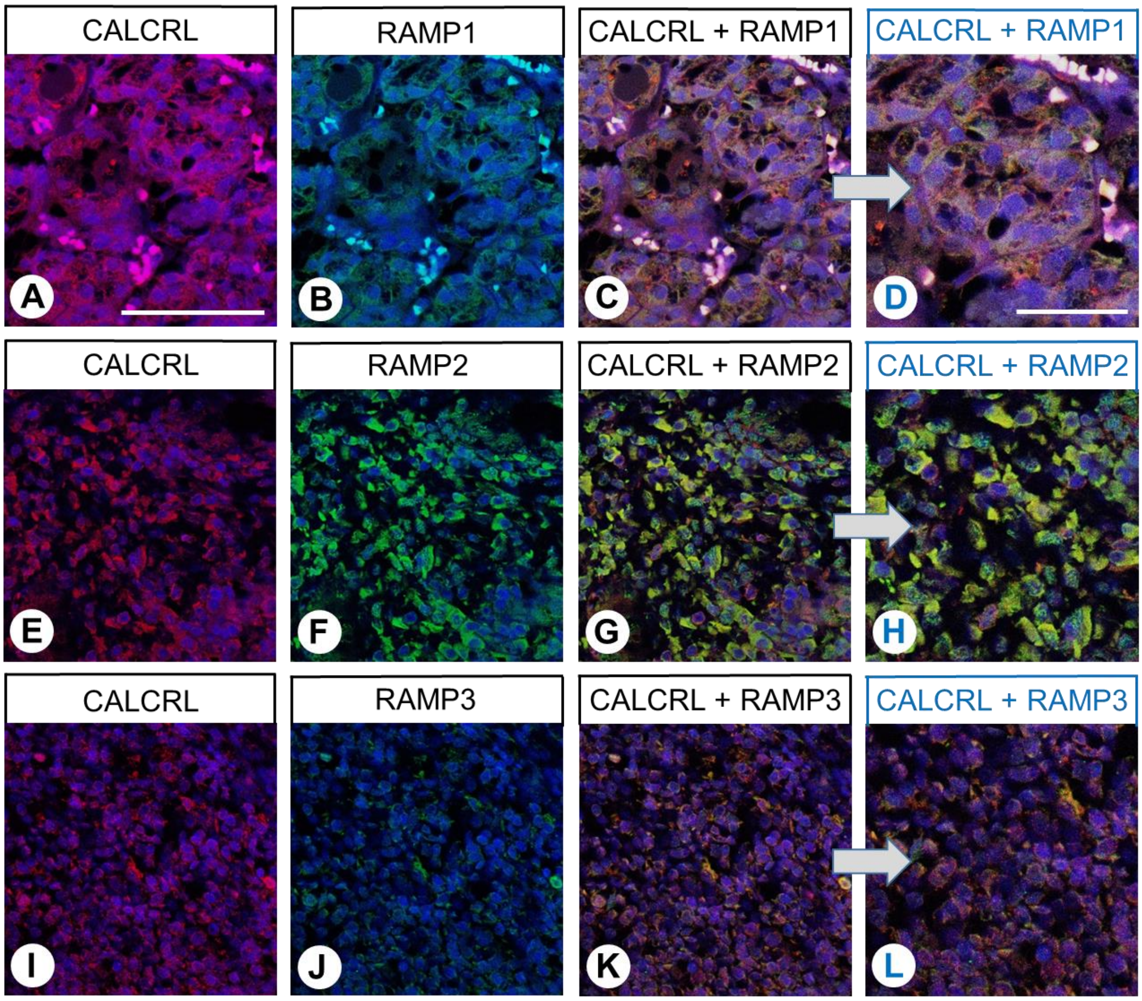

Supplemental Figure S1: Double-labelling immunohistochemical analysis of calcitonin receptor-like receptor (CALCRL) expression and the expression of receptor activity-modifying protein (RAMP) 1, RAMP2, or RAMP3 in papillary thyroid carcinoma tissue. Labelling of CALCRL was visualised using Cy3-conjugated anti-rabbit antibody (red). Labelling of RAMP1, RAMP2, or RAMP3 was visualised using Alexa Fluor 488-conjugated rabbit anti-RAMP1, RAMP2, or RAMP3 antibody (green). Overlapping expression is represented by orange/yellow colour. Blue colour represents 4′,6-diamidino-2-phenylindole (DAPI)-stained DNA. D, H and L represent enlarged sections of C, G and K. Scale bar: 100 µm (A-C, E-G, I-K); 50 µm (D, H, L).

Supplemental Figure S2

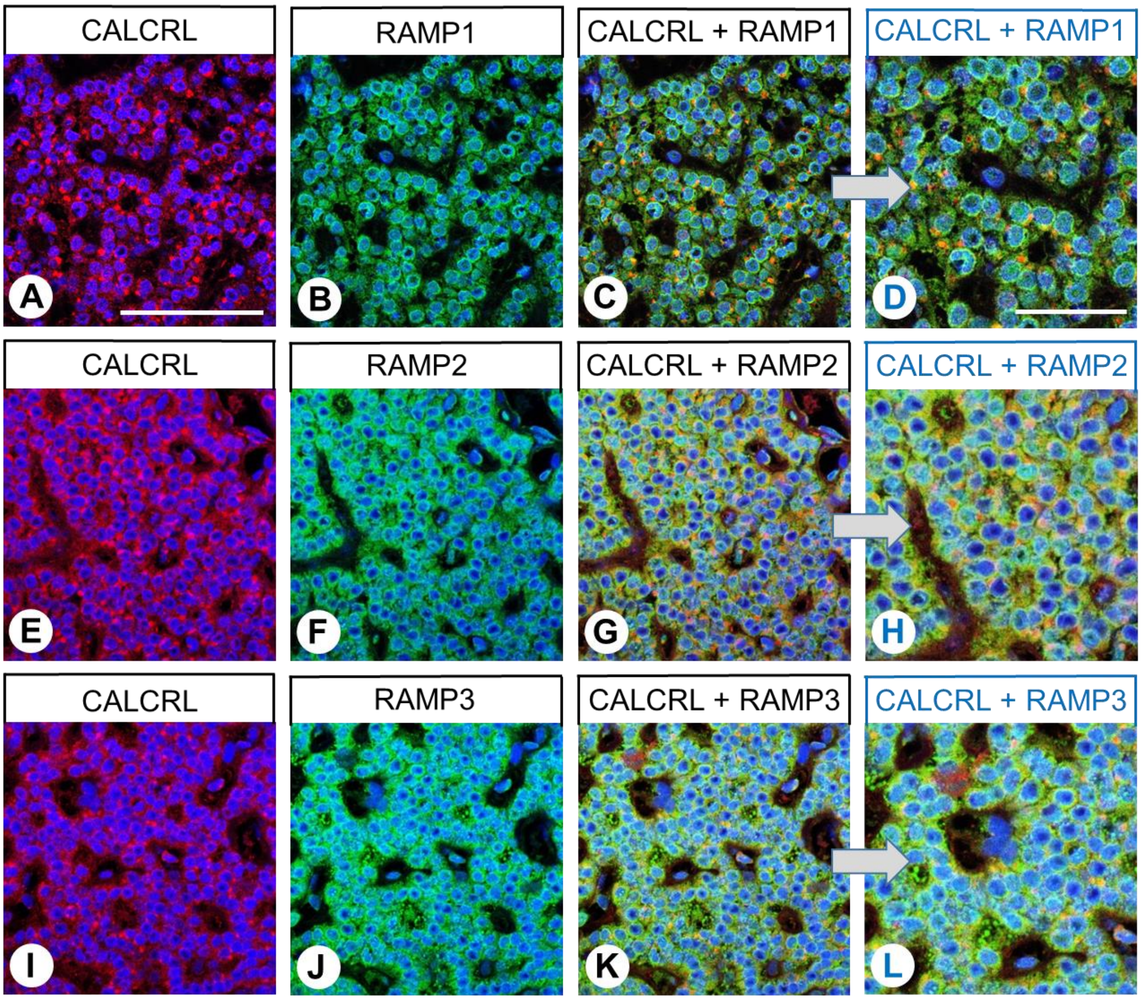

Supplemental Figure S2: Double-labelling immunohistochemical analysis of calcitonin receptor-like receptor (CALCRL) expression and the expression of receptor activity-modifying protein (RAMP) 1, RAMP2, or RAMP3 in parathyroid adenoma tissue. Labelling of CALCRL was visualised using Cy3-conjugated anti-rabbit antibody (red). Labelling of RAMP1, RAMP2, or RAMP3 was visualised using Alexa Fluor 488-conjugated rabbit anti-RAMP1, RAMP2, or RAMP3 antibody (green). Overlapping expression is represented by orange/yellow colour. Blue colour represents 4′,6-diamidino-2-phenylindole (DAPI)-stained DNA. D, H and L represent enlarged sections of C, G and K. Scale bar: 100 µm (A-C, E-G, I-K); 50 µm (D, H, L).

Supplemental Figure S3

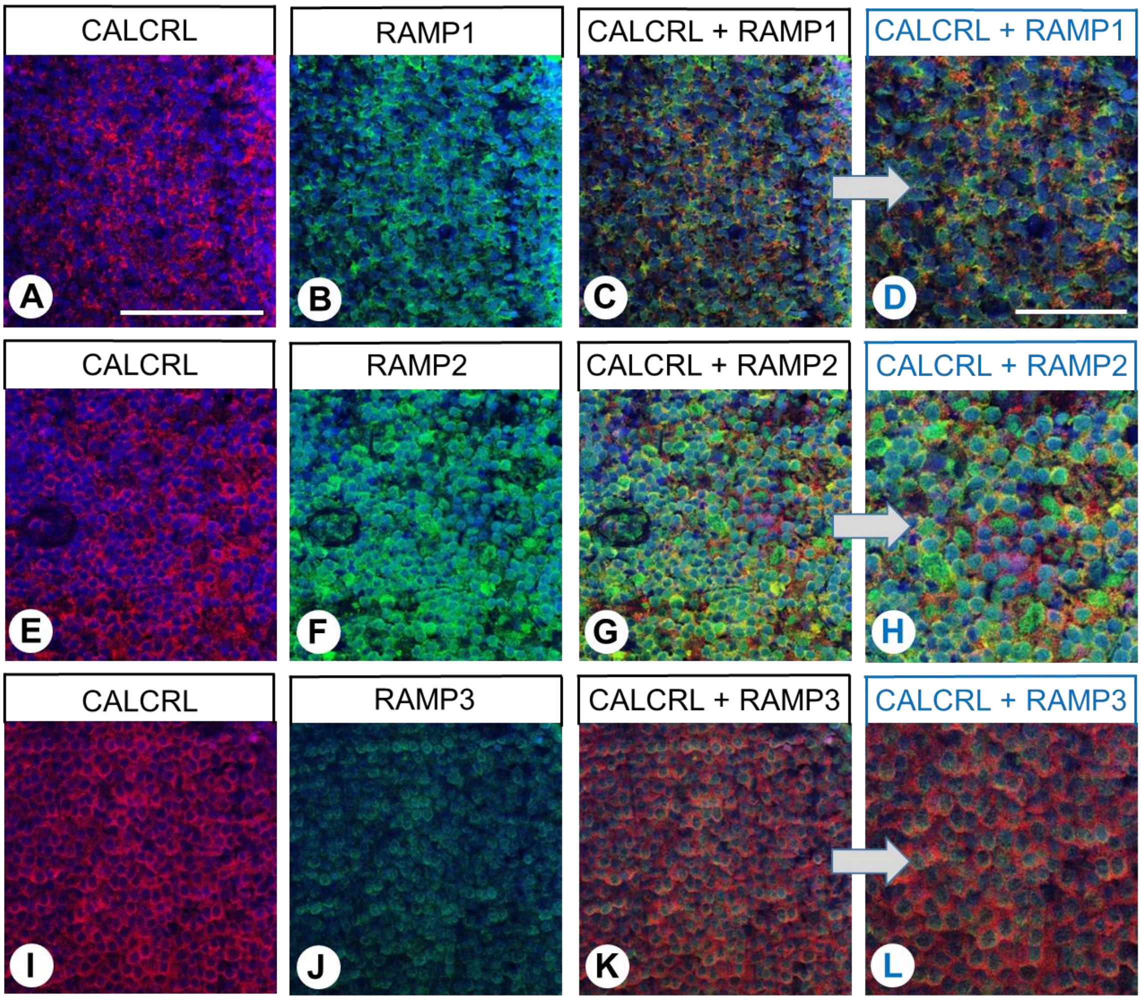

Supplemental Figure S3: Double-labelling immunohistochemical analysis of calcitonin receptor-like receptor (CALCRL) expression and the expression of receptor activity-modifying protein (RAMP) 1, RAMP2, or RAMP3 in lung adenocarcinoma tissue. Labelling of CALCRL was visualised using Cy3-conjugated anti-rabbit antibody (red). Labelling of RAMP1, RAMP2, or RAMP3 was visualised using Alexa Fluor 488-conjugated rabbit anti-RAMP1, RAMP2, or RAMP3 antibody (green). Overlapping expression is represented by orange/yellow colour. Blue colour represents 4′,6-diamidino-2-phenylindole (DAPI)-stained DNA. D, H and L represent enlarged sections of C, G and K. Scale bar: 100 µm (A-C, E-G, I-K); 50 µm (D, H, L).

Supplemental Figure S4

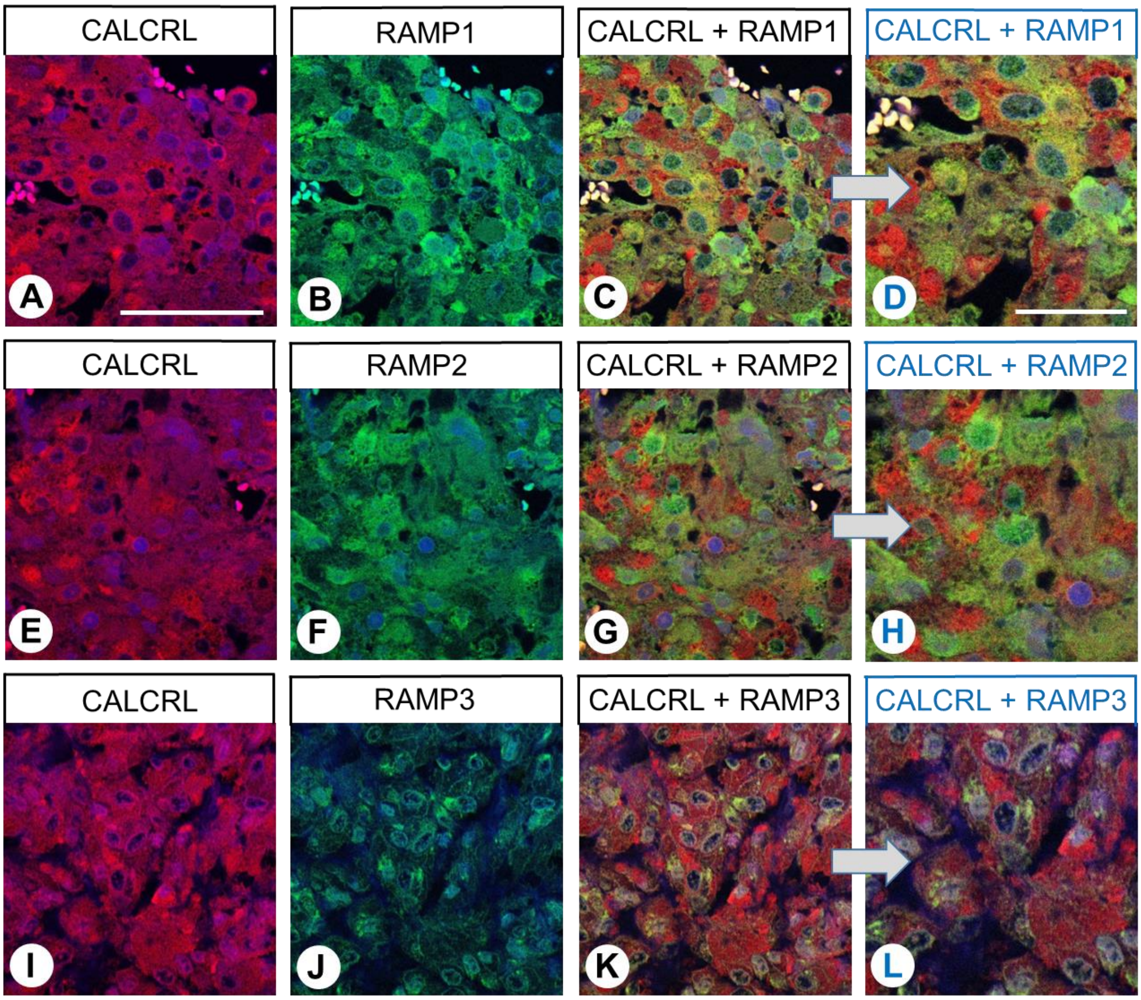

Supplemental Figure S4: Double-labelling immunohistochemical analysis of calcitonin receptor-like receptor (CALCRL) expression and the expression of receptor activity-modifying protein (RAMP) 1, RAMP2, or RAMP3 in pheochromocytoma tissue. Labelling of CALCRL was visualised using Cy3-conjugated anti-rabbit antibody (red). Labelling of RAMP1, RAMP2, or RAMP3 was visualised using Alexa Fluor 488-conjugated rabbit anti-RAMP1, RAMP2, or RAMP3 antibody (green). Overlapping expression is represented by orange/yellow colour. Blue colour represents 4′,6-diamidino-2-phenylindole (DAPI)-stained DNA. D, H and L represent enlarged sections of C, G and K. Scale bar: 100 µm (A-C, E-G, I-K); 50 µm (D, H, L).

Supplemental Figure S5

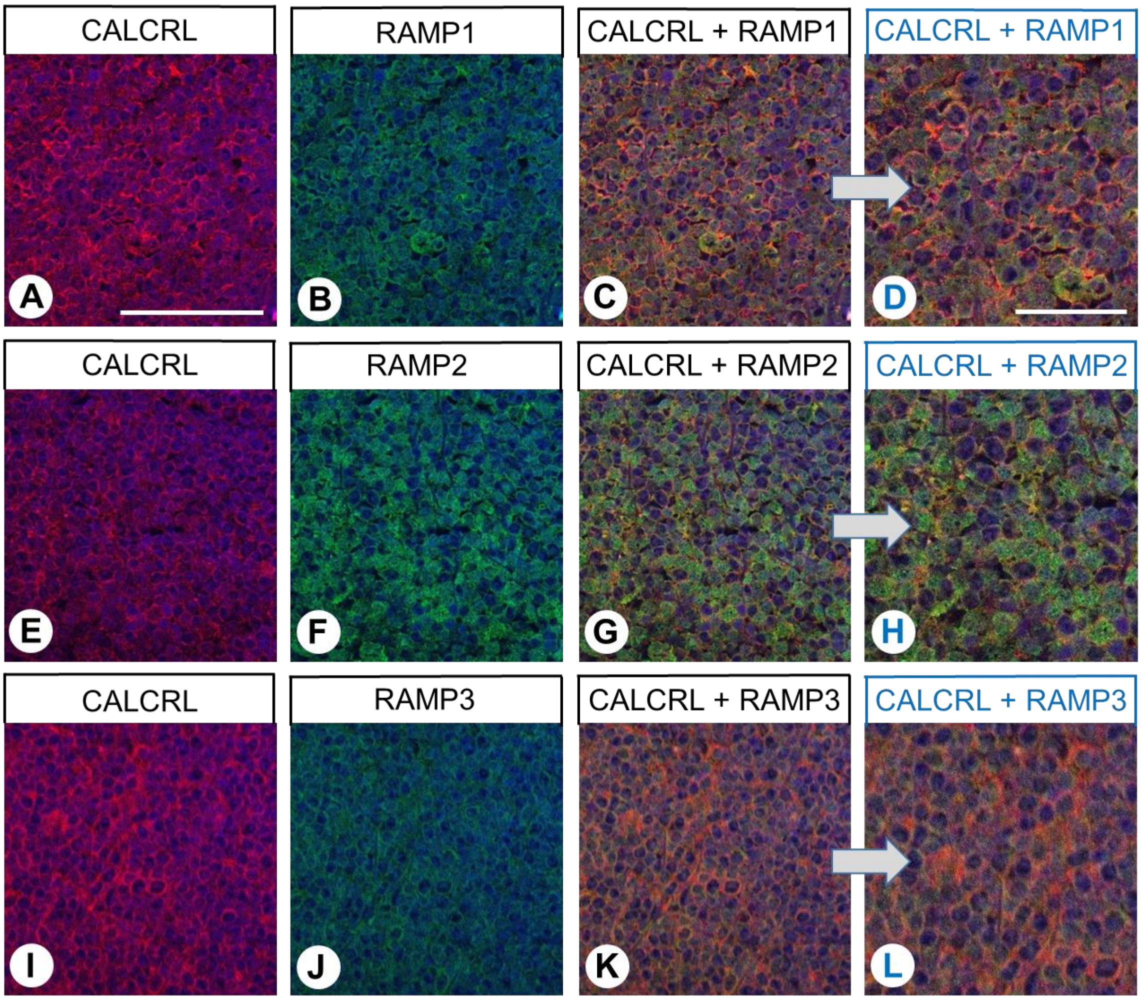

Supplemental Figure S5: Double-labelling immunohistochemical analysis of calcitonin receptor-like receptor (CALCRL) expression and the expression of receptor activity-modifying protein (RAMP) 1, RAMP2, or RAMP3 in lymphoma tissue. Labelling of CALCRL was visualised using Cy3-conjugated anti-rabbit antibody (red). Labelling of RAMP1, RAMP2, or RAMP3 was visualised using Alexa Fluor 488-conjugated rabbit anti-RAMP1, RAMP2, or RAMP3 antibody (green). Overlapping expression is represented by orange/yellow colour. Blue colour represents 4′,6-diamidino-2-phenylindole (DAPI)-stained DNA. D, H and L represent enlarged sections of C, G and K. Scale bar: 100 µm (A-C, E-G, I-K); 50 µm (D, H, L).

Supplemental Figure S6

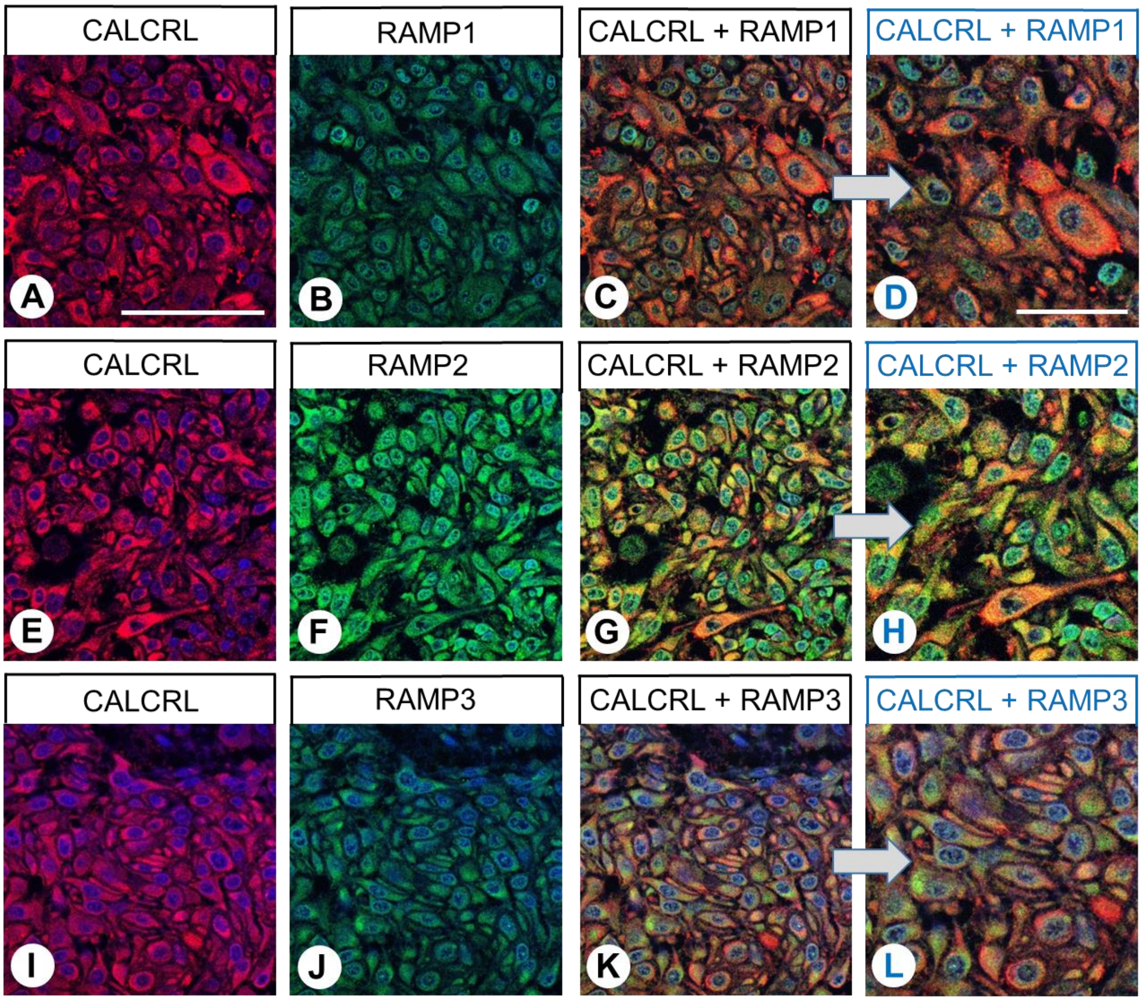

Supplemental Figure S6: Double-labelling immunohistochemical analysis of calcitonin receptor-like receptor (CALCRL) expression and the expression of receptor activity-modifying protein (RAMP) 1, RAMP2, or RAMP3 in malignant melanoma tissue. Labelling of CALCRL was visualised using Cy3-conjugated anti-rabbit antibody (red). Labelling of RAMP1, RAMP2, or RAMP3 was visualised using Alexa Fluor 488-conjugated rabbit anti-RAMP1, RAMP2, or RAMP3 antibody (green). Overlapping expression is represented by orange/yellow colour. Blue colour represents 4′,6-diamidino-2-phenylindole (DAPI)-stained DNA. D, H and L represent enlarged sections of C, G and K. Scale bar: 100 µm (A-C, E-G, I-K); 50 µm (D, H, L).

Supplemental Figure S7

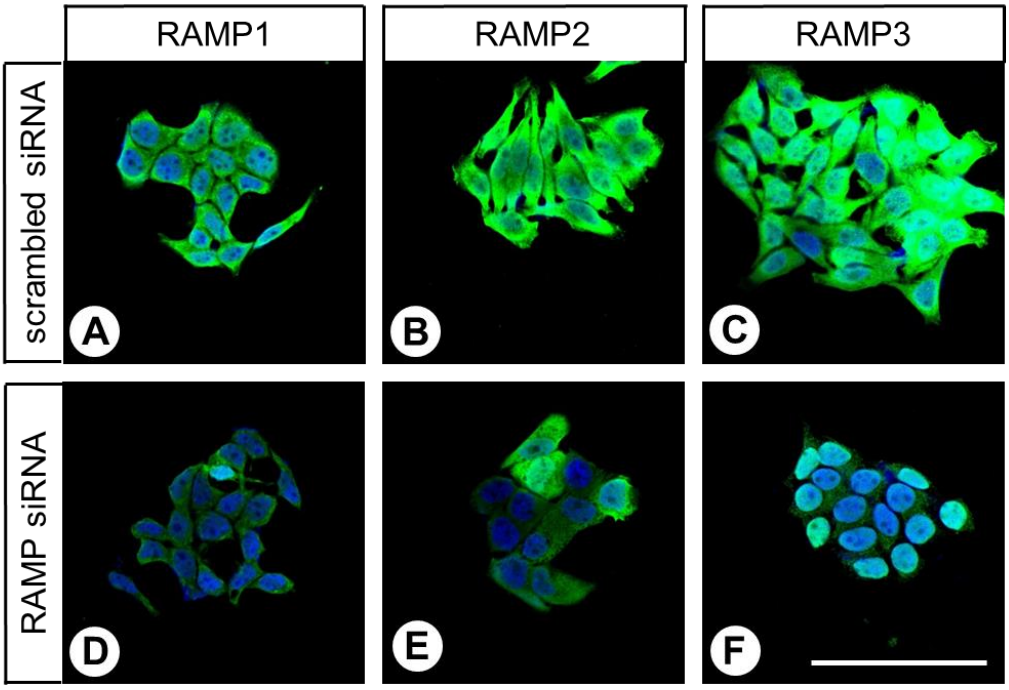

Specificity analysis of the rabbit anti-RAMP antibodies by immunocytochemistry. BON-1 cells that endogenously express all three RAMP isoforms were fixed and stained with the Alexa 488-conjugated rabbit polyclonal anti-RAMP1, RAMP2, or RAMP3 antibody (1:100 dilution; Bioss Antibodies, Woburn, MA, USA; catalogue numbers, bs-1567R-A488; bs-11971R-A488; bs-11972R-A488. To analyse antibody specificity, RAMP expression was silenced in BON-1 cells using RAMP isoform-specific siRNAs (RAMP1, sc-40894; RAMP2, sc-3678; RAMP3, sc-40896; Santa Cruz Biotechnology, Dallas, TX, USA). A scrambled siRNA was used as the negative control (Santa Cruz Biotechnology). Green colour represents the different RAMP isoforms; blue colour represents 4′,6-diamidino-2-phenylindole (DAPI)-stained DNA. Scale bar, 100 µm (A–F). All results are representative of three independent experiments.

Supplemental Figure S8

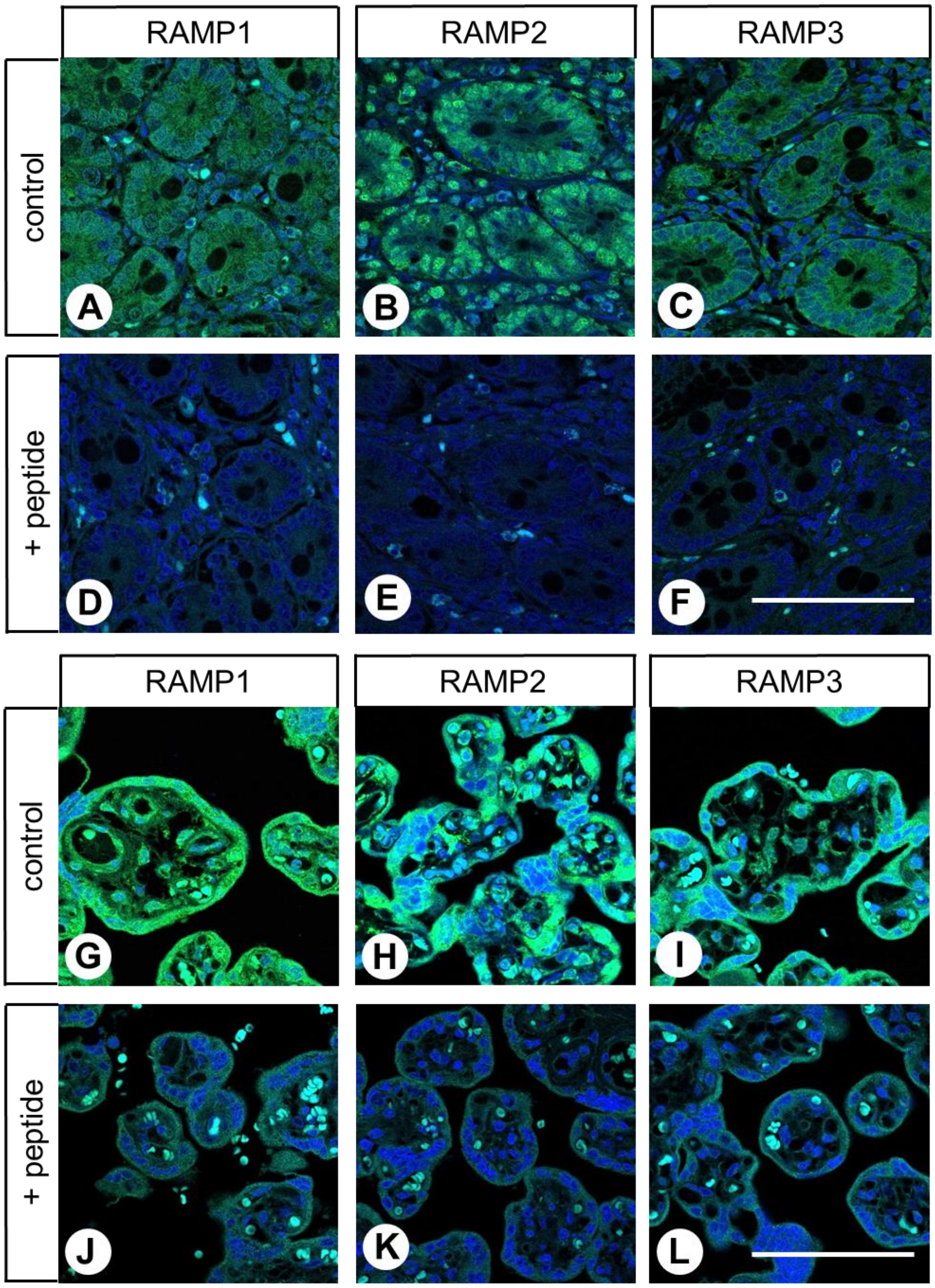

Specificity analysis of the rabbit anti-RAMP antibodies by peptide neutralisations in duodenum (A-F) and placenta (G-L) samples. RAMP antibodies were incubated for 2 h at room temperature either in the absence (control) or in the presence of 10 µg/ml of the immunising peptide (+ peptide). Labelling of RAMP1, RAMP2, or RAMP3 was visualised using Alexa Fluor 488-conjugated rabbit anti-RAMP1, RAMP2, or RAMP3 antibody (green). Blue colour represents 4′,6-diamidino-2-phenylindole (DAPI)-stained DNA. Scale bar: 100 µm
